# Vascular Dysfunction in Alzheimer’s Disease: A Prelude to the Pathological Process or a Consequence of It?

**DOI:** 10.3390/jcm8050651

**Published:** 2019-05-10

**Authors:** Karan Govindpani, Laura G McNamara, Nicholas R Smith, Chitra Vinnakota, Henry J Waldvogel, Richard LM Faull, Andrea Kwakowsky

**Affiliations:** Centre for Brain Research, Department of Anatomy and Medical Imaging, Faculty of Medical and Health Sciences, University of Auckland, Auckland 1142, New Zealand; k.govindpani@auckland.ac.nz (K.G.); lmcn644@aucklanduni.ac.nz (L.G.M.); nsmi754@aucklanduni.ac.nz (N.R.S.); c.vinnakota@auckland.ac.nz (C.V.); h.waldvogel@auckland.ac.nz (H.J.W.); rlm.faull@auckland.ac.nz (R.L.M.F.)

**Keywords:** Alzheimer’s disease, vascular dysfunction, β-amyloid, APOE, blood-brain barrier, neurovascular unit

## Abstract

Alzheimer’s disease (AD) is the most prevalent form of dementia. Despite decades of research following several theoretical and clinical lines, all existing treatments for the disorder are purely symptomatic. AD research has traditionally been focused on neuronal and glial dysfunction. Although there is a wealth of evidence pointing to a significant vascular component in the disease, this angle has been relatively poorly explored. In this review, we consider the various aspects of vascular dysfunction in AD, which has a significant impact on brain metabolism and homeostasis and the clearance of β-amyloid and other toxic metabolites. This may potentially precede the onset of the hallmark pathophysiological and cognitive symptoms of the disease. Pathological changes in vessel haemodynamics, angiogenesis, vascular cell function, vascular coverage, blood-brain barrier permeability and immune cell migration may be related to amyloid toxicity, oxidative stress and apolipoprotein E (APOE) genotype. These vascular deficits may in turn contribute to parenchymal amyloid deposition, neurotoxicity, glial activation and metabolic dysfunction in multiple cell types. A vicious feedback cycle ensues, with progressively worsening neuronal and vascular pathology through the course of the disease. Thus, a better appreciation for the importance of vascular dysfunction in AD may open new avenues for research and therapy.

## 1. Introduction

Alzheimer’s disease (AD) is a chronic neurodegenerative disorder and the predominant form of dementia [1,2]. Dementia was estimated to affect approximately 50 million people worldwide as of 2018 and this figure is expected to triple by 2050, the majority of cases being of the Alzheimer’s type [3]. AD presents with an insidious onset, with progression of symptoms over years to decades [4]. These may include the loss of memory, cognitive decline, emotional and behavioural changes, psychological impairment and loss of motor coordination [4]. From a neuropathological perspective, AD is associated with several characteristic features, the most important being progressive and extensive atrophy of the cortex and hippocampus, the deposition of insoluble β-amyloid (Aβ) within extracellular neuritic plaques and the appearance of intracellular neurofibrillary tangles (NFTs), composed of hyperphosphorylated tau protein [1,2]. AD is clinically differentiated from several other forms of dementia and graded by the appearance of the latter two pathological features. Due to an incomplete understanding of the factors underlying AD pathogenesis, a cure for the condition has been elusive. To date, only four drugs have been approved by the US Food and Drug Administration (FDA) for clinical use, but these are purely symptomatic rather than disease-modifying therapies [5,6]. Given the diversity of changes within the AD brain, it is clear that alternative mechanisms of neurological dysfunction must be considered in the design of combinatorial therapies to better address the complexity of AD pathogenesis.

**Figure 1:**
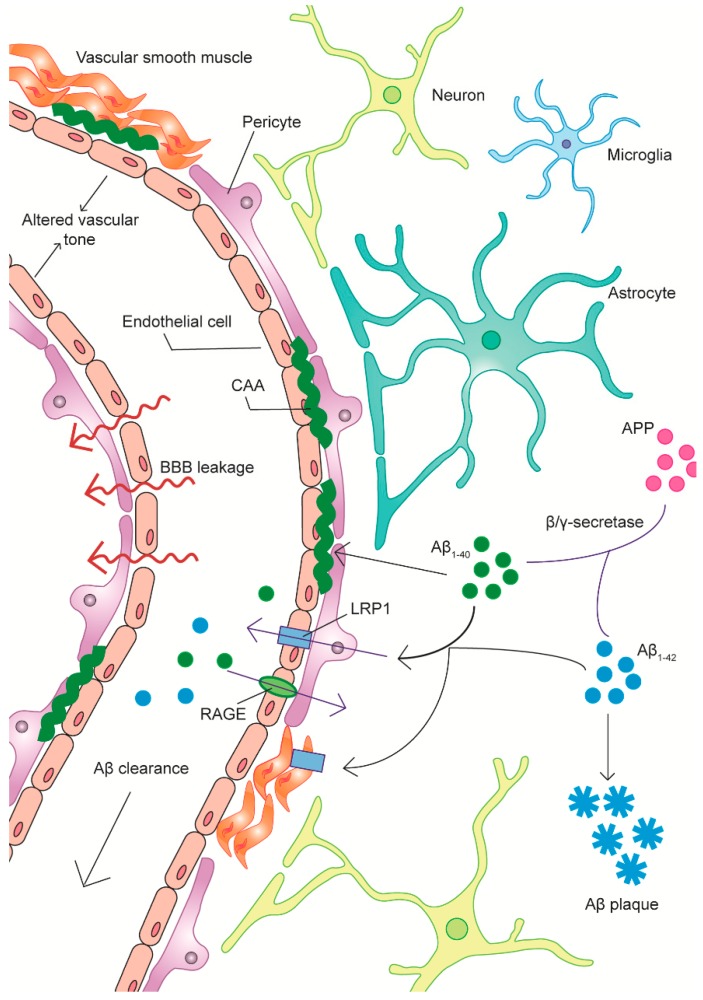
Dysregulated Amyloid-β (Aβ) Clearance in Alzheimer’s Disease (AD). The vasculature is the site of a complex amyloid-β clearance system. Pathological Aβ species, including Aβ_1-40_ and Aβ_1-42_, are generated by the cleavage of the amyloid precursor protein (APP) by the enzyme β-secretase and the subsequent cleavage of the soluble amyloid precursor protein-α (sAPPα) product by γ-secretase. Aβ binds to low density lipoprotein receptor-related protein-1 (LRP1) on the abluminal membranes of vascular cells and LRP1 mediates the internalization of the peptide by an endocytotic pathway, thus aiding in Aβ clearance and removal from the brain. The receptor for advanced glycation endproducts (RAGE), on the other hand, is involved in the transport of free Aβ from the systemic circulation into the brain. In AD, Aβ clearance mechanisms are impaired, potentially at an early stage. This includes the downregulation of LRP1 and the upregulation of RAGE in AD microvessels. Reduced Aβ clearance may contribute to Aβ deposition as parenchymal senile plaques or vascular deposits. Vascular Aβ deposition may progress to the development of cerebral amyloid angiopathy (CAA) in capillaries as well as in the smooth muscle layers of arterioles. Aβ peptides exert toxic effects on vascular cells, contribute to the dysregulation of vascular tone, induce vascular inflammation and contribute to the weakening of the blood-brain barrier. Thus, excess Aβ is involved in several mechanisms of vascular dysfunction in AD, which can also have serious consequences for disease risk and progression.

In attempting to ascertain the underlying etiology of AD, a commonly overlooked pathological aspect of the disease is the occurrence of extensive vascular dysfunction—the most apparent anatomical signs being the appearance of cerebral amyloid angiopathy (CAA) and vascular morphological and degenerative changes in affected parts of the brain [7]. Indeed, neurovascular dysfunction is ubiquitous within the AD brain and a “vascular hypothesis” of AD was suggested a quarter of a century ago based on observations of cerebral perfusion and metabolic deficits in AD patients [8,9]. In addition to these gross anatomical and physiological changes, numerous studies have reported diverse correlates of vascular cell dysfunction, including Aβ-mediated cytotoxicity, deficits in Aβ clearance, the weakening of the blood-brain barrier (BBB), aberrant immune cell recruitment and a direct vascular contribution to the pro-inflammatory state in vulnerable brain regions [10] (Figure 1). Vascular changes are an early preclinical feature of AD pathology, with changes in cortical blood flow beginning years to decades prior to the onset of clinical symptoms [11,12]. Focal decreases in blood flow in turn have an impact on amyloid clearance and neuronal metabolism [13,14]. It has been suggested in recent years that AD vascular biomarkers be incorporated into current research frameworks for the improvement of clinical AD diagnosis [15]. The symptomatic overlap between AD and vascular dementia (VD) has also been noted and both conditions respond similarly to some pharmacotherapeutic strategies [16,17]. Indeed, it has been suggested that the majority of older patients present with a mixed-dementia with characteristics of both conditions [18].

In the seminal Nun study published by Snowdon et al. in 1997, it was demonstrated that the presence of lacunar infarcts in the basal ganglia, thalamus or deep white matter causes a reduction in the neuropathological threshold (i.e., senile plaque and NFT load) required for any given grading of AD dementia [19]. These results have since been confirmed, with the additional finding that pathological comorbidity with cerebrovascular disease determines clinical presentation. It appears that senile plaque load is a predictor of cognitive deficit in combination with cerebrovascular disease, but NFT load does not appear to be a predictor of dementia severity when combined with cerebrovascular disease; NFT load is a known indicator of cognitive decline in cases where cerebrovascular disease is absent [20]. The clinical expression of AD thus appears to be significantly modified by the presence of cerebrovascular abnormalities. Given the strong association between amyloid angiopathy and atherosclerotic changes in the AD brain [21,22], it is likely that the impact of arterial amyloid deposition on vascular hemodynamics and vessel rigidity plays a significant role in arterial narrowing, increased blood pressure and the weakening of the arterial wall [22]. There is thus a growing appreciation for the link between AD risk and pre-existing cardiovascular conditions. 

There is also a significant interaction between AD pathological development, systemic vascular risk factors and genetic risk factors—the most predictive being apolipoprotein E (APOE) genotype. APOE is a protein which is important in both neural and vascular health. It is a multi-functional protein, which is involved in cholesterol transport, lipid metabolism and clearance of Aβ, among other functions [23,24]. There are 3 isoforms, APOE epsilon2 (APOE2), APOE epsilon3 (APOE3) and APOE epsilon4 (APOE4). APOE3 is the most common isoform [25]. APOE2 has been shown to be protective against AD, while APOE4 significantly increases AD risk [25,26,27] without altering the rate of cognitive decline after onset [28]. This association is even more pronounced in the presence of pre-existing chronic risk factors like diabetes [29,30,31].

Taken together, it is clear that cerebrovascular dysfunction is an area deserving of further consideration in the formulation of theories of AD pathogenesis. In this review, we present a summary of the current state of knowledge in this area and critically discuss implications for primary AD research and the search for new clinical therapies.

## 2. Cerebrovascular Alterations in Alzheimer’s Disease

### 2.1. Neurovascular Coupling Deficits and Metabolic Dysfunction

A link between impaired neurovascular coupling and AD pathogenesis was first formally proposed by de la Torre and Mussivand in 1993 [9]. Although global changes in cerebral blood flow (CBF) and glucose metabolism have been reported in the AD brain [12], several single photon emission computed tomography (SPECT) studies and arterial spin-labeling (ASL) magnetic resonance imaging (MRI) studies have demonstrated that reductions in CBF in AD are most pronounced in the temporal and parietal cortices [32,33,34,35,36,37,38,39,40,41,42,43,44,45,46,47]. These findings complement [^18^F]fluorodeoxyglucose positron emission tomography (FDG-PET) studies that reveal AD-associated reductions in glucose metabolism to be most severe in the temporoparietal cortex [48,49,50,51,52,53,54,55]. Indeed, temporoparietal CBF reduction is often considered to be a defining pathological features of AD and the utility of FDG-PET [56,57,58], SPECT [45,59,60], ASL MRI [47,61,62] and functional and perfusion MRI [63,64] in the diagnosis of AD and its differentiation from other dementias has been established. Within the parietal cortex, hypoperfusion and hypometabolism have been reported to be especially pronounced in the angular gyri [47,61] and posterior precuneus [12,47]. Reduced CBF has been noted in the frontal cortex in some studies [32,38,40,46] and hypometabolism has also been observed in this region [52,53,54], although other studies appear to dispute the occurrence of frontal perfusion deficits [33]. Interestingly, marked temporoparietal metabolic dysfunction is observed in both mild and severe AD patients, indicating that these changes may precede clinical symptoms [49]. In addition, it has been demonstrated that disease severity is associated with worsening deficits in CBF in the frontal and parietal cortices but not in the temporal cortex, suggesting a progression of blood flow abnormalities from the temporal to the frontoparietal cortices with disease progression [36]. In more established or severe AD, there is additionally greater occipital involvement [12,40,54]. Recently, 4D flow MRI studies have revealed a general reduction in total mean arterial blood flow in several major cerebral arteries as well as an increase in arterial pulsatility in AD patients [65,66].

Specific regional perfusion deficits are associated with different clinical and functional outcomes in AD patients. Cognitive performance has been correlated with CBF reductions across multiple cortical regions [12]. Additionally, asymmetric alterations in CBF have been demonstrated in numerous studies, with relevance to specific functional deficits. For instance, reduced posterior parietal CBF is correlated with the clinical presentation of apraxia in a subset of AD patients, while reduced temporal lobe CBF is correlated with memory deficits [33]. Left temporal lobe CBF appears to be more correlated with memory deficits than right temporal CBF and reductions in the left frontal, left lateral temporal and left posterior parietal regions are correlated with aphasia [33]. Results such as these underscore the importance of early vascular changes in the cortex in determining the presentation and course of AD and the contribution of asymmetric deficits to functional outcomes.

While cortical patterns of CBF dysfunction are well-attested, there is less consensus regarding subcortical structures. Some studies reported the preservation of hippocampal and subcortical blood flow and metabolism in prodromal and established AD [40,61], interesting given the centrality of hippocampal cell loss to AD pathogenesis. Posterior cingulate cortex hypoperfusion [12,61,67,68] and right anterior cingulate hyperperfusion [38] have been demonstrated in AD patients using ASL imaging and H_2_^15^O PET, but ASL and SPECT studies have inconsistently demonstrated either hypoperfusion or hyperperfusion in the hippocampus and amygdala [38,42,67,69]. Posterior cingulate hypoperfusion, in particular, is well-attested and metabolic deficiencies in this region are a feature of very early AD [70]. Hyperperfusion has been reported in the left thalamus and in limbic structures such as the right striatum, hippocampus and right amygdala in mild AD patients, potentially suggestive of compensatory effects in early AD or perhaps inflammation or some other vasodilatory stimulus [38,42]. One ASL study reported significant hippocampal hyperperfusion in early AD after accounting for grey matter loss [42]. An earlier FDG-PET study, however, reported modest decreases in caudate and thalamus glucose metabolism [54]. Cerebellar CBF is reportedly unaffected, even in later AD stages [12]. 

Differences in sub-regional perfusion deficits within these structures and hemispheric asymmetries in pathology likely contribute to some of the inconsistencies in the literature. A general weakness of such studies is in the range of disease stages represented by participants, even amongst early AD patients—some patients with preclinical signs of AD may have more severe pathology than others prior to formal diagnosis or may develop other forms of dementia. The well-established changes in temporoparietal and posterior cingulate perfusion and metabolism are likely the most reliable vascular biomarkers of risk and progression of preclinical/early AD. Another factor of concern is the lack of appropriate atrophy correction in many imaging studies, which can have significant bearing on the results of these studies [42,44]. Indeed, asymmetric perfusion deficits in the AD brain appear to closely mirror asymmetric atrophy [44]. Partial-volume corrected CBF values from many cortical regions, as measured by ASL, are much higher than uncorrected CBF values from the same brain regions [12], and appropriate volume correction may thus reduce or eliminate the statistical significance of reported changes in some sub-regions [71]. This indicates the possibility of bias in many older studies.

The elucidation of AD risk based on preclinical changes in the brain is a matter of growing interest, but relatively little is known about the earliest phases of AD pathogenesis. Given the consistency of cortical patterns of CBF and metabolic disruption in the AD brain, particularly in mild AD, and the correlation with disease severity, measures of CBF and metabolism could potentially help predict predisposition to AD prior to diagnosis. With this in mind, Okokwo et al. conducted a comprehensive study into such perfusion changes in patients with AD, as well as asymptomatic individuals with a family history of AD and those with mild cognitive impairment (MCI) with amnesia. Intriguingly, they showed that cognitively normal patients with a history of AD in the maternal line showed greater parietofrontal and hippocampal perfusion deficits than cognitively normal patients with AD in the paternal line or without a family history of AD, suggesting that some maternally-inherited factor may confer AD risk [67]. Another study utilized ASL and blood-oxygen-level-dependent functional MRI (fMRI) to show that resting CBF is elevated in the middle temporal cortex in cognitively normal APOE4-positive patients with a family history of AD and that CBF responses in these patients are diminished during functional encoding tasks compared with low-risk individuals [72]. Thus, regional CBF and glucose metabolism measured by standard medical imaging techniques could be useful as biomarkers of AD risk or early pathological changes in the disease prior to the onset of clinical symptoms, and this could perhaps aid in the assessment of potential preclinical therapies. Indeed, at-risk individuals may show metabolic deficits in temporoparietal cortical regions [73]. FDG-PET has been used successfully to track regional declines in metabolic rate over time, mirroring cognitive decline, and changing glucose metabolism in AD-affected brain regions may thus be a good biomarker of AD progression over time [74]. With regards to CBF changes, one SPECT study was able to differentiate between healthy controls, patients who presented with signs of impending AD, patients who had just been diagnosed with AD and patients with established AD [75]. It was shown that prior to clinical AD onset, hypoperfusion in the hippocampus, amygdala, posterior cingulate gyrus and left anterior thalamus precedes deficits in the temporoparietal cortex [75]. SPECT has also been used to correlate the Braak stage progression of AD with perfusion changes in various brain regions, revealing the appearance of deficits in anterior middle temporal, subcallosal, posterior cingulate and cerebellar perfusion between the entorhinal and limbic stages, and then large deficits in posterior temporoparietal perfusion between the limbic and neocortical stages of the disease [39]. The progression of deficits has also been correlated with decreases in cerebrospinal fluid (CSF) Aβ and increases in CSF tau prior to the onset of AD. Abnormal Aβ downregulation in the pre-AD CSF appears to be associated with the onset of temporoparietal CBF reduction and this hypoperfusion then worsens with the onset of abnormal CSF tau upregulation [12]. High CSF phospho-tau (p-tau) and total tau in the healthy brain have been correlated with reduced CBF in frontotemporal regions [76]. The elucidation of such stereotyped and progressive changes in pre-AD patients and through the course of the disease could aid in differential diagnosis at an early stage of disease progression and aid in preclinical prediction of disease risk, potentially allowing for early intervention. It has been shown that brain Aβ load in early AD is associated with decreased blood flow in various brain regions [77]. Aβ_1-40_ application reduces resting CBF in APP transgenic mice [78] and vascular Aβ deposition in APP/PS1 mice brings about regional CBF reductions [79]. Inter-individual variations in Aβ load may have a significant effect on patterns of perfusion changes in AD, with high Aβ load being associated with longitudinal increases in perfusion in some brain areas and decreases in others [80]. Regional CBF even in the healthy brain may show reductions in the presence of Aβ deposition [81] and metabolic disturbances may be observed in these brain regions [82,83,84]. Thus, correlations between CBF reduction and other molecular biomarkers of AD are well attested.

Although APOE genotype is considered to be the most significant genetic risk factor for AD, the link between APOE genotype and CBF dysfunction is controversial. Many studies have failed to show a correlation between this factor and the severity or distribution of perfusion or metabolic deficits in AD [55,67,75,85,86], with one study even demonstrating dose-dependent increases in frontotemporal metabolism [87]. This may be consistent with the finding in previous studies that APOE status, while modifying AD risk, does not affect the rate of disease progression following AD diagnosis [28]. However, many others have demonstrated reduced CBF and metabolism across multiple cortical regions in APOE4-positive AD patients compared with non-carriers [88,89,90,91,92,93]. Once again, it is important to consider the impact of AD stage and the evolving nature of these deficits, the importance of volumetric correction and appropriate analysis in imaging studies [89], the use of different imaging techniques, the effect of APOE4 gene dose, correction for confounding factors like age and sex, and the impact of patient sample sizes. APOE4 homozygous patients in some studies reportedly present with greater perfusion and metabolic dysfunction than heterozygotes [87,93] but also with greater volume loss in the temporal cortex [94]. Given the inconsistencies between studies examining patients with established AD, it is perhaps more instructive to look at the contribution of APOE4 to pre-clinical risk of AD. Individuals with a family history of AD and positive for APOE4 display pronounced metabolic deficits in several cortical regions, in a pattern similar to that observed in AD patients [67,95,96]. Cognitively normal APOE4 carriers display more significant perfusion deficits with age across multiple sub-regions of the frontal, temporal, parietal and cingulate cortices compared with non-carriers, which could contribute to increased AD risk with age [71,84,97]. Cognitively normal APOE4 carriers also appear to display increased CBF in the left lingual gyrus and the cuneate nucleus, particularly in older carriers [97]. Interestingly, hyperperfusion is observed in the left anterior cingulate cortex in younger APOE4 carriers, while hypoperfusion occurs in older carriers [97], potentially indicating very early compensatory changes followed by the development of more pronounced deficits and CBF reduction. Indeed, younger APOE4 carriers appear to display greater activation in hippocampal and default network circuits during memory encoding, reflected in BOLD fMRI measurements from these regions, and this over-activation gives way to significantly reduced activation with age [98,99]. Thus, the very early contribution of APOE4 to neuropathological processes may be recapitulated by CBF and cerebral metabolic changes through the lives of carriers prior to the clinical appearance of AD. It is also likely that APOE4 status contributes to the heterogeneity in imaging-based measures of early CBF changes in AD.

The causes of perfusion deficits in AD are likely numerous. However, changes in blood flow are believed to be due in large part to changing patterns of vascular innervation with neuronal loss [100]. Cognitive reserve may result in the preservation of cognitive function until a critical mass of neuronal loss has been achieved [101]. Deficits in neurovascular coupling may thus become pronounced prior to the onset of clinical symptoms. The cholinergic hypothesis is based on the observation that post-mortem AD brains exhibit severely decreased cholinergic innervation in several brain regions, in particular the temporal cortex and hippocampus [102]. This is believed to play a role in the cognitive and behavioral deficits characterizing the disease, alongside several other pathological contributors. The cholinergic-vascular hypothesis posits that the significant cholinergic neuron loss observed in AD results in decreased vasodilatory tone in innervated vessels, due to the fact that cholinergic neurons play a key role in the maintenance and control of vascular tone in affected brain regions [103,104]. Cholinergic nerve terminals projecting from the basal forebrain are closely associated with arterioles in the frontal, parietal and temporal cortices and cholinergic innervation to cortical vessels is greatly reduced in AD [104]. Acetylcholine (ACh) functions as a vasodilator through muscarinic receptors on vascular smooth muscle cells (vSMCs) and decreased ACh tone likely contributes to greater baseline vasoconstriction in affected areas [105]. It is important to note that other neurotransmitters like glutamate and noradrenaline have also been demonstrated to act as vasoactive agents, either indirectly through intermediate vasoactive factors or directly by acting on contractile vascular cells like pericytes and vSMCs [106,107,108]. Thus, the loss of glutamatergic and noradrenergic innervation in AD [109,110] could similarly disrupt neurovascular coupling mechanisms. Many excitatory neurotransmitter systems, including the cholinergic system, may be coupled to vascular changes either directly or indirectly through GABAergic interneurons or glial cells [105,111,112,113]. Glial cell dysregulation is a key pathological feature of AD [114] and this likely also plays a role in neurovascular coupling deficits. Thus, preclinical perfusion deficits are likely the result of early loss or remodeling of innervation in the presenile brain, resulting in metabolic dysfunction and potentially contributing to a feedback loop involving neuronal death in affected brain areas. Reduced blood flow in vulnerable areas is associated with reduced clearance of Aβ, potentially resulting in enhanced neurotoxicity and deposition in these regions [13].

Numerous other vasoactive signaling mechanisms are believed to become dysfunctional in AD but these will not be reviewed exhaustively. The vasoactive effects of Aβ and the effects of neuroinflammatory mediators on vasoactive changes will be covered in brief in later sections. We will also review the possible mechanisms of vascular amyloid deposition and touch on how this may contribute to impaired vascular contractility and reductions in vSMC and pericyte number, which may have important implications for perfusion in the AD brain.

### 2.2. Vascular Morphology and Angiogenesis

It is well established that vascular abnormalities are common in the AD brain at the macrostructural level. Several early qualitative studies reported distortions in small arterioles and capillaries in the AD brain, particularly in the hippocampal region and temporoparietal cortex. These vessels were often described as being tortuous, kinking, looping, twisting, spiraling or forming bundles and knob-like structures [115,116,117,118,119]. Such dementia-related changes in arteriole structure may be accompanied by the thinning of vessel walls and the loss of smooth muscle and elastic tissue [116]. While changes in brain volume and parenchymal tissue loss may influence vascular architecture, features like vessel lengthening and the formation of “wicker-like” networks point to the active influence of other factors on angiogenesis [116]. Such structural changes may exert a significant impact on local blood flow, with looped vessels and abnormal structures contributing to increased vascular resistance and disturbing the overall hemodynamic state of the local vascular network [115,120]. It has also been found that the density of “string vessels”—non-functional capillary remnants mostly composed of connective tissue and lacking in endothelial cells—is significantly increased in the gray matter of the AD brain [121,122,123] and also increased, albeit to a lesser extent, in the gray matter of brains from patients without dementia but exhibiting amyloid pathology [121]. String vessel density appears to be greatest in brain regions with high Aβ load [123]. A similar increase in string vessel density, greater than two-fold, also occurs in the white matter, along with an increase in string vessel length and string length as a proportion of total vessel length [124]. The exact reason for increased string vessel formation in the AD brain remains unknown. Increases in string vessel density as a result of endothelial degeneration are likely to be closely linked to decreased perfusion and metabolic dysfunction in the AD brain. However, string vessels may also be the result of aberrant angiogenesis and changing patterns of vessel coverage. It has been shown in the healthy brain that transient endothelial tube sprouting and retraction can leave behind these structures and that pathologically affected tissues display more of these outgrowths [125].

Angiogenic processes may be disturbed in AD, possibly contributing to the process of string vessel formation. Despite reports of sometimes marked reductions in vascular density in the AD cortex, hippocampus and basal forebrain [115,118], there is evidence also of upregulated angiogenesis. It has been reported that vascular sprouts composed of endothelial processes are readily discernible in tissue from advanced AD cases [118]. One study reported increased vascular density within the AD hippocampus, with an increase in angiogenic vessels positive for integrin *α*v*β3* [126] and another demonstrated a significant increase in vessel density in the AD cortex [127]. Rather than being contradictory, this may be indicative of the remodelling of surviving vascular networks in the AD brain. Supporting this idea, young APP23 mice display denser vascular networks around amyloid plaques that are associated with truncated blood vessels [128]. Another possibility is that angiogenesis in the AD brain may be aberrant, with new vessels being poorly formed and prone to premature regression due to vascular cell death and growth factor downregulation [129,130]. It has been shown in both AD patients and AD mouse models that angiogenic vessels may differ from patent vessels in several respects, including the presence of abnormal cellular morphology, aberrant branching and disturbed basement membrane and junction formation, and angiogenesis may be localized [128,131,132]. Neoangiogenesis in this context may in fact be detrimental to vascular dynamics and the integrity of the BBB, despite causing increases in vascular density. It is also important to note that many older studies in particular have failed to account adequately for tissue atrophy and AD stage—there are likely changes in the extent and impact of angiogenesis with worsening pathology. Aberrant angiogenesis and endothelial death may be coterminous, with the relative contributions of these processes changing over the course of the disease. 

Several markers of angiogenesis, including vascular growth factors, show disturbed expression profiles in AD. Vascular endothelial growth factor (VEGF), a potent and specific mediator of endothelial cell proliferation [133], is released by numerous cell types, including neutrophils [134,135,136], onto the endothelium to stimulate angiogenesis [137]. VEGF also plays a role in the regulation of BBB permeability, increasing the leakiness of the microvascular wall [138]. VEGF expression changes are well attested in AD patients. Capillary VEGF expression is reduced within the temporal cortex, hippocampus and brainstem in AD patients [139]. Serum levels of VEGF and transforming growth factor-β1 (TGF-β1) are reduced in AD patients [140,141,142], with lower levels correlated with greater cognitive deficits [140], potentially suggestive of a contribution of angiogenic deficits to the evolution of the disease. VEGF and TGF-β were reportedly found at heightened levels in CSF samples from AD patients [143]. Lower CSF VEGF levels are correlated with smaller hippocampal volume and ventricular expansion in individuals with high brain Aβ load [144] and the heightened CSF VEGF levels observed in AD could thus represent a protective response. VEGF release from natural killer (NK) cells and lymphomononuclear cells isolated from AD patients is also impaired and Aβ_1-42_ suppresses NK cell VEGF secretion [145]. Aβ_1-42_ also competitively antagonizes VEGF binding to VEGF receptor-2 (VEGFR-2) on endothelial cells [146], which could contribute to the anti-angiogenic properties of Aβ. The VEGF-165 isoform reportedly binds to Aβ with high affinity, resulting in its sequestration into senile plaques in the AD brain and potentially reducing VEGF availability at the vasculature [147,148]. In addition to preventing vessel sprouting, VEGF inhibition in spontaneous and implanted tumours in mice has been shown to cause the regression of existing blood vessels, leaving behind string vessel structures—such a process could also be relevant to AD [149]. Considering all of this and the reported neuroprotective functions of VEGF against hypoxic [150] and excitotoxic damage [151] and amyloid aggregation [148], the stimulation of VEGF synthesis and release could represent a therapeutic strategy in the prevention of neurovascular dysfunction and other pathological processes in AD. Indeed, VEGF supplementation in APP/PS1, PDGF-hAPP^V717I^ and TgCRND8 mice results in cognitive improvement, increased angiogenesis, decreased endothelial apoptosis and reductions in amyloid and p-tau load [152,153,154]. Interestingly, insulin has been shown to counteract the effect of Aβ_1-42_ on VEGF synthesis in NK cells from AD patients [145]. Given the presence of widespread insulin signaling defects in the AD brain [155], this could be relevant to neurovascular dysfunction and the heightened AD risk conferred by diabetes. One study reported the upregulation of VEGF in perivascular astrocytes and in extracellular deposits close to the vasculature but this was more associated with vessels exhibiting a high degree of cerebral amyloid angiopathy (CAA) [156]. Aβ injection in mice also stimulates increased VEGF synthesis in astrocytes and microglia [157]. Hypoxia is known to stimulate angiogenesis through the upregulation of VEGF synthesis [158]. Astrocytes, in particular, upregulate VEGF synthesis in response to hypoxia, in a process mediated by hypoxia-inducible factor-1 (HIF-1) [159]. Therefore, high astrocyte VEFG around amyloidogenic AD vessels may be a protective response to more pronounced hypoxia in these tissues. Indeed, HIF-1α expression is upregulated in AD microvessels [160]. In the developing retina, it has been shown that endothelial filopodia migrate along astrocyte mesh networks secreting VEGF and astrocytic upregulation of VEGF in response to hypoxia could thus conceivably cause abnormal vessel branching in AD [161].

There are several other factors that could potentially contribute to dysregulated angiogenesis in the AD brain. Mesenchyme homeobox 2 (MEOX2), a regulator of vascular cell differentiation during angiogenesis, is downregulated in the AD cerebral vasculature, likely contributing to impaired angiogenesis [162]. Matrix metalloproteinase-9 (MMP-9) expression also appears to be reduced in AD microvessels, alongside the upregulation of tissue inhibitor of matrix metalloproteinase-1 (TIMP-1) [163]. MMP-9 is involved in endothelial migration and basement membrane remodeling during angiogenesis [164]. Transferrin receptor upregulation in the cerebral vasculature in some AD patients may be consistent with its role in cell proliferation [165].

Thus, there is abundant evidence to suggest that impaired angiogenesis in the AD brain is driven at least in part by defective growth factor availability and signaling mechanisms. The expression of vascular growth factors and the occurrence or suppression of angiogenesis may also be influenced by vascular Aβ load and more pronounced hypoxia due to perfusion deficits might activate perivascular astrocytes and alter their angiogenic signaling [156]. Indeed, it has been hypothesized that hypoxia is a key driver of aberrant angiogenesis in AD and increasing hypoxia with progressive pathological impairment may cause stress-related increases in angiogenesis [166]. A direct role for Aβ has also been confirmed in the regulation of angiogenesis and vessel branching [157,167,168]. The expression of growth factors like VEGF is regulated by a variety of pro-inflammatory cytokines that are themselves affected in AD [169]. Given the complex nature of the molecular pathways associated with angiogenesis and the evolving profile of vascular dysfunction in the AD brain, it is perhaps not surprising that the nature and extent of angiogenic changes in previous studies has been inconsistent. Thus, there is a need for studies clarifying this phenomenon and its importance in the development and progression of human AD.

### 2.3. The Blood-Brain Barrier

#### 2.3.1. The Neurovascular Unit

Neurovascular coupling and other cerebral vascular functions are primarily mediated at the level of the neurovascular unit (NVU), an anatomical and functional unit comprising multiple vascular and other CNS cell types in close apposition. The NVU is composed of endothelial cells that form the walls of the blood vessel, vSMCs and perivascular pericytes that attach to a basal lamina and wrap tightly around the vessel, interneuron terminals and astrocyte end-feet in close association with the vessel wall, and the extracellular matrix (ECM) [170]. The components of the NVU are functionally linked in an intimate and efficient manner via gap junctions, adhesion molecules and the local release of vasoactive agents and neuromodulators [170]. The release of such agents in particular by interneurons and astrocytes allows for the rapid and precise modulation of blood flow as well as vascular cell function and homeostasis in response to local metabolic demand [171]. Blood flow modulation is not a passive process but is tightly controlled by contractile changes in pericytes and vSMCs in response to local neuronal modulation. Endothelial cells, tightly packed together via tight junctions, form the semi-permeable and highly selective BBB, essential in the maintenance of normal brain ion balance and homeostasis and functioning as a protective barrier against systemic infection and inflammatory processes [172]. Together, the NVU and BBB contribute to the complex process of amyloid clearance in the healthy brain. This involves several specialized transporters in the endothelial cell membrane, including LDL-family receptors and transporters for carrier proteins to which Aβ can bind like apolipoprotein J (APOJ), APOE and α2-macroglobulin [173,174,175]. Thus, vascular cells play an important physiological role in the regulation of brain Aβ levels, both in terms of actively transporting Aβ into vessels and in regulating perfusion and removal from the brain. BBB dysfunction is widespread in the AD brain, to the extent that it has been suggested as one of the primary vehicles of neurovascular dysfunction in the disease [176].

In the previous section, endothelial cell loss was alluded to as a potential mechanism underlying the reduced vessel densities reported by some studies in the post-mortem AD brain. In addition to the unusual vascular architectures observed in the disease, there is a marked disturbance in vascular cell morphologies within the neurovascular unit. In some studies, endothelial cells in the AD cerebral cortex were reported to be swollen, with significant cellular atrophy [177,178,179]. Hypertrophic changes, collagen accumulation and detachment have been noted in the endothelial basement membrane [177,178,179]. Collagen deposition is observed around capillaries in the AD brain [178] and medial and adventitial collagen deposition has also been noted in the walls of arterioles [179]. Basement membrane thickening may be associated with the deposition of amyloid fibrils as aggregates in vessel walls [180]. A relationship between endothelial atrophy and amyloidosis was also reported, with capillaries exuding amyloid fibrils being among the first to degenerate [177]. However, capillary degeneration is more frequent and widespread than vascular amyloid angiopathy, potentially indicating a longer-term degenerative process in the AD cerebral microvasculature that might be partially independent of amyloid toxicity [177]. Endothelial cell degeneration appears to occur at the level of the individual cell, with cells adjacent to those undergoing degenerative changes often showing no signs of dystrophy themselves [178]. Lipofuscin accumulation within endothelial cells might also point to membrane, mitochondrial or lysosomal damage. Endothelial cells in the AD brain may show decreased mitochondrial content [178,181] and the abnormal upregulation of pinocytotic vesicles in the cytosol in a manner inversely proportional to mitochondrial loss [178]. Pinocytotic upregulation could point to increased permeability of the BBB and mitochondrial loss could interfere with the function of ion channels and active transport systems reliant on ATP [178]. Changes in basement membrane thickness and composition could have an effect on both BBB permeability and vessel stiffness, and basement membrane detachment is seen in some vessels [178,182].

Pericyte and vSMC degeneration are also frequently-described features of AD. Morphologically, pericytes can be seen to undergo alterations in the AD brain, with cytosolic debris observed with electron microscopy [179]. Like endothelial cells, a large subset of AD pericytes are reported to contain extensive cytosolic lipofuscin deposits [178]. Pericyte loss in AD can be widespread and severe, with one study reporting as many as 35% of capillaries in the AD brain to be affected [178]. Pericyte number and coverage are significantly reduced in the AD cortex and hippocampus [183,184] and a recent electron microscopy study revealed extensive pericyte degeneration in the parietal, visual and acoustic cortices and in the hippocampus [119]. This study also noted significant subcellular mitochondrial alterations in pericytes, endothelial cells and perivascular astrocytes, including enlarged and dystrophic microglia and a reduction in mitochondrial number per cell [119]. In APP^sw/0^/Pdgfrβ^+/−^ mice, which overexpress Swedish mutant APP and are deficient in platelet-derived growth factor receptor-β (PDGFRβ), pericyte loss occurs gradually with age and this is associated with an elevation of Aβ levels and CAA, significant tau hyperphosphorylation in the cortex and hippocampus and early neuronal loss in these regions [185]. The reduction in pericyte number observed in human AD could thus have severe implications for neurodegeneration. The exact mechanisms underlying pericyte loss in AD are yet to be elucidated but a link with amyloid angiopathy has emerged based on studies in mice and in human cell cultures (see Section 2.4.2). Pericyte loss may itself be induced by inflammatory activation (see Section 2.6), with the release of MMP-9 for instance by activated pericytes having been shown to cause enhanced pericyte migration [186]. Indeed, low density lipoprotein receptor-related protein 1 (LRP1) expression in brain pericytes is upregulated during inflammation, which might further contribute to Aβ-induced pericyte loss [187]. Pericytes play an important role in the maintenance of BBB integrity and pericyte loss has been directly implicated in BBB breakdown in AD and other disorders like amyotrophic lateral sclerosis (ALS) [183,188]. An illustrative example of the importance of pericytes in BBB maintenance can be found in pericyte-deficient mutant mice, in which the BBB is far more permeable to molecules of both high and low molecular mass [189]. Pericyte loss in the human AD precuneus and parietal white matter is correlated with increased fibrinogen infiltration, indicative of BBB breakdown [190].

In addition to AD-associated mortality and dysfunction in the vascular cells of the neurovascular unit, these cells may themselves exert neurotoxic effects on neurons and other cell types in AD. The idea that a dysfunctional vasculature could directly contribute to neurotoxicity is not new. In in vitro co-cultures, AD microvessels cause neuronal cell death through apoptotic or necrotic pathways [191]. AD microvessels have been shown to release significantly higher levels of nitric oxide (NO) into the surrounding parenchyma [192]. In older patients, this NO release may reach neurotoxic levels [192]. Thrombin synthesis is also highly upregulated in microvascular endothelial cells in the AD brain and this is reflected in high CSF thrombin levels [193]. Thrombin is known to possess neurotoxic properties at high concentrations, triggering neuronal death through a variety of pathways and potentially playing a role in the worsening of amyloid and tau pathology [193]. As will be discussed in Section 2.4.3, there is also evidence that vSMCs in AD can synthesize and release soluble Aβ, which may then contribute to parenchymal plaque formation. Endothelial cells may also be capable of APP synthesis and secretion [194] and cerebral microvessels have been shown to exude amyloid fibrils in AD [195]. Importantly, vascular cells are also mediators of neuroinflammation, releasing a variety of pro-inflammatory molecules when activated (see Section 2.6). Such findings suggest once again that rather than simply being victims of neuronal and glial dysfunction in AD, vascular cells may themselves contribute directly to neurodegenerative processes. Thus, neuro-glial and vascular dysfunction likely interact with each other and generate progressively worsening degenerative feedback loops.

#### 2.3.2. BBB Breakdown—A Leaky Barrier

The permeability of the BBB is primarily regulated by the tightness of the junctions between closely apposed endothelial cells forming the vessel wall. This barrier allows for the regulation of solute exchange between the blood and brain parenchyma while precluding the exchange of several large molecules and pathogenic agents from the systemic circulation. Importantly, the BBB also regulates the migration of immune cells across the vessel wall. Thus, the BBB is essential in the regulation of brain homeostasis, while maintaining its status as an immunologically-privileged organ.

It was reported as early as 1982 that the AD cortical neuropil is immunoreactive for serum proteins like albumin and globulins, particularly in the vicinity of amyloid plaques which themselves stain strongly for these factors but not necessarily at sites of overt vascular leakage [196]. The increased presence of hemoglobin peptides in the AD cerebellum was also reported as being a likely consequence of BBB leakage [197]. Various other vascular factors like prothrombin [198], fibrinogen [183,199] and immunoglobulin G (IgG) [183] have also been noted in the AD cortex and hippocampus, further confirming the aberrant permeability of the BBB in the disease. More recently, MRI studies have demonstrated significant global BBB leakage within the cortical gray matter in patients with early AD [200,201]. Reduced CBF is correlated with cortical BBB leakage [201] and the extent of cortical BBB leakage is correlated with cognitive decline [200]. BBB leakage has also been shown in the hippocampus in patients with MCI, without any concomitant changes in hippocampal volume [202]. The occurrence of BBB leakage prior to significant atrophic change suggests that BBB breakdown may occur in the early stages of the disease or even preclinically.

The barrier function of the endothelial layer is regulated by tight junctions (TJs)—intercellular barriers consisting of a network of proteins including claudins, junction adhesion molecules (JAMs), TJ-associated MARVEL proteins (TAMPs) (including occludin) and several other components [203]. Electron microscopy reveals that capillaries in the AD hippocampus and parietal, visual and acoustic cortices display a reduced number and length of tight junctions [119]. There is evidence for the pathological dysregulation of tight junctions by Aβ and this will be covered in Section 2.4.4. In particular, disturbed expression of occludin, claudin-1, zonula occludens-1 (ZO-1) and zonula occludens-2 (ZO-2) have been reported in response to Aβ in vitro, as well as in mouse models of AD [127,204,205]. Hypoxic conditions and oxidative stress have also been shown to cause occludin and ZO-1 downregulation in cerebral and dermal endothelial cells in vitro, possibly involving extracellular signal-regulated kinase-1/2 (ERK1/2) activation and VEGF signaling [206,207]. Occludin downregulation was enhanced in the presence of glucose deficiency, suggesting the contribution of glucose to this stress-mediated pathway [206,208]. In vitro, oxidative stress also causes the downregulation of cadherins in dermal endothelial cells [208]—cadherins are important in the formation of adherens junctions. Given the extent of perfusion and metabolic deficits in the AD brain and the widespread hypoxic conditions, it can be speculated that this might have a direct impact on tight junction and adherens junction formation and stability in existing and angiogenic vessels. The increase in BBB permeability in AD may also involve an increase in endothelial transcytosis associated with pericyte loss, as observed in pericyte-deficient mice [189]. Interestingly, these pericyte-deficient mice also display subtle changes in junctional width and distribution pattern [189]. The increase in pinocytotic vesicles observed in AD endothelial cells in ultrastructural studies may be related to increased transcytotic transport [178]. 

The pathological migration of immune cells across the BBB is believed to play a role in AD neurodegeneration. Activated microglia resident in the AD parenchyma may have serious pathogenic consequences, as a result of the inflammatory and phagocytic pathways mediated by these cells [209,210]. Activated microglia likely play a protective role in the AD brain, being found around Aβ plaques and possibly being involved in their immunological isolation from the surrounding parenchyma [209,210]. However, microglial degeneration is apparent in the AD brain and both the over- and under-activation of microglia may contribute to the disease process depending on the pathological context [209,210]. Other innate immune cells may similarly exert protective or deleterious effects and their uncontrolled migration across the BBB and into the parenchyma could also contribute to neurodegeneration through the over-activation of pro-inflammatory pathways and the secretion of neurotoxic agents [211]. This may also have consequences for the integrity of the BBB itself. Monocytes, macrophages and lymphocytes have been shown to migrate at a higher rate through the weakened BBB in AD and macrophages interact closely thereafter with senile plaques, assume perivascular positions or infiltrate the vessel wall [212]. In particular, these cells appear to be associated with sites of TJ damage, revealed by the absence of ZO-1 [212]. Macrophage migration has similarly been associated with tight junction damage in HIV-1 encephalitis and HIV-1-associated dementia [212,213,214]. Monocytes and macrophages are believed to transition into microglia after entry into the brain [215] and unregulated microglial precursor infiltration through a damaged BBB could have serious consequences for the degenerative process. Neutrophil trafficking across the BBB is increased in 5xFAD mice and 3xTg-AD mice and this appears to be related to the vascular upregulation of adhesion molecules like vascular cell adhesion molecule-1 (VCAM-1), intercellular adhesion molecule-1 (ICAM-1), E-selectin and P-selectin [216]. In AD mouse models and in the AD brain, Aβ plays a role in the recruitment of immune cells to the BBB and transmigration across the endothelial layer—this will be discussed in Section 2.4.4.

APOE genotype appears to influence BBB breakdown in AD patients. APOE4 homozygotes with AD have thinner capillary basement membranes in the prefrontal cortex than APOE3 homozygotes and non-carriers with AD [217] and increased leakage of plasma proteins into the cortical neuropil has been measured in APOE4-positive AD patients compared with non-carriers [198]. In an in vitro BBB model reconstituted with APOE4-positive mouse astrocytes, tight junction deficits were observed, including reduced phosphorylation of occludin [218]. Human APOE4 knock-in mice also exhibit increased BBB impairment [218]. APOE4 carriers with AD, and to a lesser extent APOE3 carriers, appear to be far more prone to pericyte dysfunction than non-carrier AD patients, resulting in an accelerated pericyte degenerative phenotype and proportional worsening of BBB breakdown [219]. This was shown to be associated with increased intracellular accumulation of MMP-9 and cyclophilin A (CypA) in endothelial cells and pericytes in AD patients homozygous for APOE4 [219]. It has previously been shown in transgenic mice that the pro-inflammatory CypA-MMP9 pathway in pericytes is associated with pericyte death and BBB breakdown and that the APOE3 genotype may actually be protective against this CypA-MMP9-mediated pathway through the interaction of astrocyte-secreted APOE3 with the LRP1 receptor [220]. Meanwhile, unlike APOE3, APOE4 appears not to suppress this pathway, resulting in greater cerebrovascular injury [220]. Indeed, BBB breakdown is also observed prominently in APOE-knockout mice [221]. CypA and MMP-9 were also shown to be upregulated in CSF samples from older cognitively normal APOE4 carriers and this was correlated with a heightened plasma albumin quotient, indicating potentially increased BBB breakdown in these individuals [222]. This is consistent with the contribution of APOE4 to preclinical risk of AD dementia. Indeed, APOE4-positive human brain pericytes in vitro are far more prone to Aβ_1-40_ toxicity than APOE3- or APOE2-positive pericytes [223].

#### 2.3.3. Glucose Metabolism

Early studies reported the downregulation of glucose transporters (GLUTs) in cortical and hippocampal AD microvessels [224,225]. Glucose transporter-1 (GLUT1) and glucose transporter-3 (GLUT3) in particular have been reported to exhibit decreased expression in the temporoparietal cortex [226], with GLUT1 reductions also seen in AD frontal cortex microvessels [227] and in the AD hippocampal endothelium [228]. A recent study also demonstrated a significant downregulation of GLUT1 in brain-derived circulating endothelial cells from patients with mild AD [229], suggesting that this process may precede many of the clinical and pathological manifestations of the disease, much like the metabolic dysfunction observed in pre-symptomatic individuals who go on to develop AD. FDG-PET has revealed deficits in forward glucose transport in the temporal and frontal cortices of AD patients and this could be related to this observed glucose transporter downregulation [230]. GLUT downregulation could thus contribute to the neuronal metabolic defects observed in the disease. However, the observed transport deficit may not entirely account for the decreased metabolism observed in these brain regions [230].

Vascular cells may also be directly affected by GLUT downregulation in combination with amyloid pathology. It was recently demonstrated that GLUT1-deficient APP^Sw/0^ mice overexpressing APP exhibit a range of pathological changes compared with controls, including accelerated amyloid deposition, cognitive-behavioral deficits, diminished Aβ clearance with elevated Aβ, perfusion deficits, increased neurodegeneration, capillary degeneration, decreased vessel length and the accelerated breakdown of the BBB [231,232]. However, it was shown that only endothelial GLUT1 reduction is related to BBB degenerative changes and not GLUT1 downregulation in astrocytes [231]. These mice, of course, were deficient in GLUT1 from the embryonic stage and GLUT1 has been shown in zebrafish to be essential to BBB development– the knockdown of GLUT1 in zebrafish results in endothelial cell loss from an early stage of development and the early downregulation of tight junction proteins [233]. Genetically-induced GLUT1 knockdown/knockout may have different implications for the BBB than the presumably gradual GLUT1 downregulation observed in AD—indeed, adults with rare genetic GLUT1 deficiencies do not appear to exhibit such early symptoms of AD [234], although age-related changes and interaction with Aβ load later in life have not been studied. It is possible that vascular cell proliferation and BBB integrity would be affected in similar ways in GLUT-1 deficient individuals in the presence of pathological levels of Aβ. GLUT1 receptors could thus be a target in the treatment of early neurometabolic dysfunction and BBB disruption in early AD.

### 2.4. Amyloid-Related Vascular Pathology and BBB Clearance Deficits in AD

#### 2.4.1. A Link between Vasoactive Dysfunction and Aβ Pathology

Aβ is a peptide generated by the proteolytic cleavage of amyloid precursor protein (APP), by β-secretase-1 (BACE1) at the N-terminus and γ-secretase at the C-terminus [235]. The amyloid cascade hypothesis proposes that these Aβ plaques are the main drivers of AD pathogenesis, causing neurotoxicity and cell death [236].

In recent years, there has been mounting evidence suggesting a link between vascular dysfunction and Aβ pathology. A study by Thomas et al. [237] provided the earliest evidence that Aβ possesses vasoactive properties, showing that the application of Aβ to rat aorta segments caused the vessels to constrict. Furthermore, pre-treatment with Aβ reduced the amplitude of ACh-mediated vasodilation. This study concluded that Aβ interacts with endothelial cells, causing the overproduction of free radicals that induce vascular changes. Aβ-induced vasoactivity was blocked by superoxide dismutase (SOD), an endogenous antioxidant, further confirming the involvement of oxidative stress pathways. An ensuing study elaborated on this discovery, showing that Aβ enhanced the action of the potent vasoconstrictor endothelin 1 (ET-1) and that Aβ_1–40_ was the peptide most effective at eliciting this response [238]. It was later shown that this Aβ-induced vasoconstriction is mediated by a pro-inflammatory signaling pathway, beginning with phospholipase A2 (PLA2) secretion and eventually resulting in the metabolism of arachidonic acid into proinflammatory eicosanoids [239]. Aβ can directly stimulate cyclooxygenase-2 (COX-2) in human cerebral blood vessels, stimulating the secretion of vasoactive prostaglandins [240]. vSMCs have also been shown to display hypercontractility and reduced responsiveness to vasoactive cues with Aβ administration [241] and this could contribute to changes in CBF in early AD. Indeed, Aβ_1-40_ administration in the mouse brain results in reduced resting CBF [242]. Like vSMCs, pericytes appear to respond to Aβ by contracting, potentially also contributing to CBF changes in the AD brain [243]. Thus, Aβ may exert a direct vasoactive role with consequences for cerebral perfusion in AD.

Further studies reaffirmed such mechanisms in vivo, establishing stronger links to AD. The first group to do so investigated Aβ-induced oxidative stress in an AD mouse model [244]. They found that transgenic mice overexpressing APP exhibited selectively impaired endothelium-dependent regulation of microcirculation in the neocortex. Additionally, mice simultaneously expressing superoxide dismutase 1 (SOD1) or that had SOD1 topically applied to the cerebral cortex, showed no such endothelial dysregulation. This showed that Aβ-induced free radical overproduction by endothelial cells could also occur in vivo and therefore may be contributing to the CBF reduction seen in AD. A subsequent study elaborated on this discovery, showing that transgenic mice overexpressing APP exhibit impaired cerebrovascular autoregulation [245]. Additionally, it was demonstrated that transgenic mice overexpressing APP and Aβ exhibited attenuated functional hyperemia in the neocortex in response to vibrissal stimulation. These results were recapitulated by the topical application of Aβ_1-40_ to the neocortex, but were not observed in response to Aβ_1-42_ [246]. CBF reductions in response to Aβ_1-40_ have been shown to be dose-dependent in mice [78]. Aβ_1-40_ was also found to attenuate CBF increases induced by endothelium-dependent vasodilators, but not endothelium-independent vasodilators, strengthening the idea that endothelial cells mediate Aβ_1-40_-induced vasoconstriction. These responses to Aβ_1-40_ were counteracted by the superoxide scavengers SOD and manganese (III) tetrakis (4-benzoic acid) porphyrin (MnTBAP). Another study, employing similar techniques, showed that both Aβ_1-40_ and Aβ_1-42_ caused a dose-dependent enhancement in the amplitude of CBF reductions produced by the vasoconstrictor U-46619 [242]. 

There is considerable discourse over the exact mechanisms involved in these Aβ-induced vasoactive processes [247]. It is established, as stated earlier, that Aβ clearance in the human CNS is decreased in AD [13]. As it stands, there are a few prominent hypotheses that seek to explain these findings. The Aβ clearance hypothesis posits that the accumulation of Aβ is due to an imbalance between its production and clearance. The claim is that Aβ clearance directly into the blood is likely the most prominent pathway, however the perivascular route and Aβ degradation are acknowledged as alternative mechanisms for clearance [248]. A neurovascular hypothesis has previously been suggested, presenting the idea that neurovascular coupling deficits, blood vessel regression, reduced CBF and neurovascular inflammation are caused by a combination of impaired Aβ clearance across the BBB, aberrant angiogenesis and cerebrovascular degradation. This likely culminates in the BBB becoming compromised, thereby altering the normal neurochemical environment of the brain parenchyma and causing neuronal dysfunction, injury and loss [249]. Finally, the two-hit vascular hypothesis suggests that initial damage to the brain microvasculature caused by chronic risk factors like heart disease, hypertension or diabetes initiates non-amyloidogenic vascular events (hit one). Primarily, these are localized CBF reductions and BBB dysfunction, which cause ischemic and toxicity-induced neuronal death, respectively. BBB collapse possibly reduces Aβ clearance from the brain and this reduced perfusion induces Aβ overproduction, resulting in Aβ accumulation in the brain (hit two) [250]. This uncleared Aβ potentially reinforces the existing vasculotoxic and neurotoxic effects produced by hit one, forming a pathological feedback cycle that eventually manifests as AD [250]. The specific contributions of different cerebrovascular cell types to Aβ clearance deficits will be presented in this section.

#### 2.4.2. Aβ-Endothelial Cell Interactions

Several mechanisms have been implicated in the reduced Aβ clearance seen in AD. The majority are related to the dysfunction and collapse of the BBB, facilitating Aβ deposition in the brain and around the vasculature [248]. The mechanism most frequently cited is the downregulation of LRP1, the major endothelial cell surface receptor responsible for the transcytosis of Aβ out of the brain across the BBB [251]. It was shown that this occurs via an isoform-specific LRP/Aβ protein-protein interaction. The study in question demonstrated that this interaction favours the clearance of Aβ_1-40_ over Aβ_1-42_ and vasculotropic mutant Aβ. This was validated in a transgenic mouse line expressing a mutant form of Aβ_1-40_ that binds LRP1 with low affinity, resulting in lower levels of Aβ clearance compared with controls. Furthermore, this study found that a proteasome-dependent form of LRP1 degradation in the endothelium was promoted by Aβ, offering a possible mechanism for these findings [252]. The effects of LRP1 downregulation were later reaffirmed, with a further study showing that a mixture of phosphorothioate antisenses against LRP1 mRNA caused decreased LRP1 expression, both in vitro in isolated mouse brain microvessels and in vivo when injected into mice [253]. In vivo, this lead to decreased Aβ_1-42_ clearance across the BBB, increased Aβ_1-42_ accumulation within the brain parenchyma and cognitive impairment [253]. Another later investigation shed light on the mechanisms underlying these findings, demonstrating that phosphatidylinositol-binding clathrin assembly protein (PICALM) is integral to the LRP-mediated transcytosis of Aβ across the BBB. This study found that PICALM levels in isolated cortical microvessels from late-stage AD brains were reduced compared with controls and that this was inversely correlated with Aβ load and clinical dementia rating [254]. Importantly, this study also showed that the Aβ-LRP1 interaction triggers a conformational change in LRP1 that enhances PICALM binding, initiating PICALM/clathrin–dependent endocytosis of the Aβ-LRP1 complex [254]. Silencing PICALM and clathrin heavy chain significantly inhibited Aβ_1-40_ transcytosis and PICALM+/− mice exhibited greater retention of Aβ_1-40_ and Aβ_1-42_ within the brain [254]. Furthermore, when a single-nucleotide polymorphism in the PICALM gene known to be protective in AD was expressed in induced pluripotent stem cell (iPSC)-derived endothelial cells, it was found that the protective allele resulted in 72–78% higher expression of PICALM mRNA and protein and 120% higher clearance of Aβ than the AD-associated allele. Thus, PICALM may have potential as a future AD drug target [254]. Further research elaborated on the established proteasomal-dependent degradation of LRP1 [252]. It was shown that silencing the gene for MEOX2, a regulator of vascular differentiation with low expression in AD, caused a substantial reduction in LRP1 expression by brain endothelial cells. LRP1 synthesis was unaffected however, as evidenced by unchanged levels of its immature form. Moreover, when the cells were treated with MG132, a proteasomal-dependent LRP1 degradation inhibitor, it resulted in the normalization of LRP1 levels. Their findings in MEOX2+/− mice took this a step further, showing substantial Aβ_1-40_ retention in the brain and an 80% reduction in the efflux of Aβ_1-40_ across the BBB compared with their wild-type counterparts. They concluded that the low expression of MEOX2 seen in AD may act to promote the proteasomal degradation of endothelial LRP1 in the brain, leading to Aβ accumulation [162]. Overall, these findings offer extensive support for the hypothesis that endothelial LRP1 expression and function become impaired in AD, reducing Aβ clearance across the BBB and causing it to accumulate within the brain, thus promoting cognitive decline.

It is also important to note that LRP1 does not exist solely as a membrane-bound receptor. A soluble form that circulates within the plasma—soluble LRP (sLRP)—has also been identified [255]. Research has shown that sLRP is also likely implicated in AD. A study by Sagare et al. [256] demonstrated that sLRP acts as a peripheral ‘sink’ for Aβ, binding free Aβ and thereby regulating its metabolism. Individuals with AD exhibit reduced plasma levels of sLRP compared with controls and furthermore, their oxidized sLRP content is significantly higher [256]. Taken together, these results indicate that sLRP is an important Aβ binding protein in human plasma, retaining it in the vessel lumen and preventing its entry into the brain, with this function possibly being impaired in AD.

However, LRP is not the only protein implicated in the breakdown of the BBB in AD, with the receptor for advanced glycation end-products (RAGE) also playing a crucial role. The earliest evidence for the effect of RAGE on mechanisms involved in AD development was provided by Yan et al. [257]. They demonstrated that RAGE is capable of binding Aβ_1-40_ and Aβ_1-42_, thereby mediating the interaction of Aβ with endothelial cells and neurons, leading to oxidative stress and microglial activation. A later study tested the binding of a radiolabeled Aβ homologue to human endothelial cells in an in vitro AD model and showed that apical binding was significantly downregulated when an anti-RAGE antibody was applied [258]. Additionally, the study found that transfected cells overexpressing RAGE and scavenger receptor (SR) type A bound the homologue and then carried out apical-to-basal transcytosis. Such findings provided early evidence for the involvement of RAGE in Aβ transport [258]. Indeed, it was subsequently shown that RAGE-expressing cells in the vasculature are responsible for the transcytosis of Aβ from the lumen, across the BBB and into the brain. This was shown by infusing transgenic mice with a radiolabeled Aβ homologue, resulting in its RAGE-mediated, concentration-dependent uptake into the brain. Moreover, it was found that this process caused an increase in proinflammatory cytokine expression and triggered the production of ET-1, with the latter causing CBF reductions [259]. A later study by Donahue et al. [260] sought to compare RAGE and LRP1 expression between AD and control hippocampi using fluorescent immunohistochemistry and Western blot. Control hippocampi displayed high levels of neuronal RAGE staining, however staining on the microvasculature was weak. The opposite pattern was true for LRP1. Conversely, AD hippocampi exhibited low levels of neuronal RAGE staining but strong microvascular staining and the pattern of LRP1 staining was similarly reversed. Western blot demonstrated that both RAGE and LRP1 were present at higher concentrations in AD hippocampi compared with controls, with the latter possibly due to LRP1 co-localization with senile plaques. These results indicate that the hippocampal distribution patterns of these receptors change in AD. Furthermore, these changes occurred in a manner suggesting that these receptors promote the retention of Aβ from the circulation within the brain parenchyma, in agreement with previous findings (Figure 1).

#### 2.4.3. Aβ-Vascular Smooth Muscle Cell and Aβ-Pericyte Interactions

It is important to note that, in addition to endothelial cells, LRP1 is expressed by several other cell types in the neurovascular unit, including pericytes and vSMCs. There is a wealth of evidence suggesting that these cells are involved in Aβ uptake and degradation themselves and also the transport of Aβ from the brain parenchyma to endothelial cells for degradation (Figure 1). Urmoneit et al. [261] were the first to show that cerebral vSMCs are involved in Aβ_1-40_ and Aβ_1-42_ clearance in vitro, rapidly internalizing Aβ from the CSF through receptor-mediated endocytosis and sequestering it into endosomes and lysosomes for degradation. They were also able to show that serum lipoprotein, APOE and LRP receptors are essential for this endocytotic process [261]. An LRP1-mediated endocytic mechanism was later confirmed in vivo. In this study, it was demonstrated that LRP1 antagonism in vSMCs caused decreased uptake and degradation of Aβ and LRP1 knock-out in vSMCs in APP/PS1 mice caused increased Aβ and plaque aggregation [262]. This suggests that vSMCs may also play an important role in the regulation of Aβ levels in the brain and that the downregulation of LRP1 and Aβ degrading proteases like neprilysin in AD vSMCs may result in Aβ tissue accumulation and vascular deposition [263].

A key amyloid-related pathology observed in AD, and a direct consequence of impaired Aβ clearance, is CAA—the deposition of Aβ in the walls of cerebral and leptomeningeal blood vessels, including arteries, arterioles, capillaries and veins. CAA is a common pathology associated with ageing and can be divided into multiple subtypes based on the specific amyloid protein involved. One study showed CAA in approximately 80% of AD patients, with moderate to severe pathology in about 25% of patients [264]. Higher CAA load is correlated with several deleterious outcomes, including increased risk and frequency of ischemic lesions, cerebral haemorrhage and cerebral arteriosclerosis [264,265,266]. In addition, the co-occurrence of CAA and hypertension in AD patients may increase the risk of cerebral infarction [265]. Hypoperfusion and the loss of vascular contractility in AD results in reduced interstitial fluid drainage, resulting in greater Aβ deposition—this in turn further contributes to vessel stiffening, exacerbating the problem [267]. The deposition of Aβ in vessel walls is associated with the thickening of vascular walls and the constriction of blood vessels [265], the deterioration of vSMCs [268] and the degeneration of vessels [269]. Around single vessels, amyloid pathology may range from thin layers of Aβ within the smooth muscle layer to large perivascular plaques [270]. This deposition interferes with neurovascular coupling mechanisms, both by restricting the contractile potential of vessels and by causing associated neuronal synapses and microglia to become dysfunctional. CAA has been observed in AD mouse models, including mice with the Dutch and Iowa vasculotropic APP mutations [271]. In these models, it has been determined that Aβ deposition is the result of impaired clearance across the BBB. Aβ-induced vSMC deterioration could also contribute to the further loss of Aβ clearance capacity in vessels, further exacerbating plaque deposition within the vessel wall. CAA occurs primarily within the tunica media smooth muscle layer in supra-capillary vessels and this is believed to be related to LRP1-mediated Aβ clearance deficits in AD [262]. In both AD-derived human vSMCs and in AD mouse models, it was reported that the transcription factors serum response factor (SRF) and myocardin (MYOCD) are significantly upregulated within vSMCs [272], resulting in Aβ clearance deficits through the expression of sterol regulatory element binding protein-2 (SREBP2) and the subsequent downregulation of LRP1 [273]. Another study reported that vSMCs exposed to saturating levels of Aβ in vitro upregulate LRP1 and low density lipoprotein receptor (LDLR) [274] but this could simply be a compensatory response to acute exogenous Aβ application. The modulation of LRP1 could represent a direction for therapeutic intervention in AD.

It was suggested early on that APOE is essential in the LRP1-mediated internalization of Aβ by vSMCs and Aβ is internalized in complex with APOE [261]. Indeed, both APOE3 and APOE4 have been reported to induce cytosolic Aβ accumulation in a dose-dependent manner, with APOE4-induced intracellular Aβ deposits being more stable and more slowly degraded [275]. The expression and activity of neprilysin, a key enzyme involved in Aβ degradation, are reduced in AD in both the vasculature and parenchyma [263,276]. Interestingly, APOE4-positive patients display even more significant reductions than non-carriers [276], while APOE2-positive brain neprilysin levels are least affected [277]. Low neprilysin levels are associated with risk of vascular Aβ deposition and CAA in the AD brain [276] and neprilysin protects vSMCs from Aβ toxicity in vitro [277]. With regards to Aβ uptake, all three isoforms of APOE, in association with high-density lipoprotein (HDL), were shown to reduce Aβ_1-42_ uptake by vSMCs in vitro [278]. In vivo also, APOE4 was shown to disrupt the BBB clearance of Aβ in mice, with the binding of Aβ to APOE4 apparently favoring slower internalization by very-low-density lipoprotein receptors (VLDLRs) rather than the rapid LRP1-mediated internalization; however, APOE2 and APOE3 in complex with Aβ are cleared equally by both LRP1- and VLDLR-mediated processes [279]. Supporting this, APOE2 and APOE3 increase Aβ uptake by Chinese hamster ovary (CHO) cells but APOE4 does not [280]. Thus, while APOE is indeed essential in the LRP1-mediated uptake of Aβ by vSMCs, APOE4 appears to be detrimental to Aβ clearance by these cells. This is consistent with the association of the APOE4-positive genotype with parenchymal Aβ deposition as well as the lack of reported CBF deficits in pre-symptomatic APOE3 carriers. The appearance of APOE4-induced intracellular Aβ deposits is correlated with oxidative stress in vSMCs in vitro [275]. APOE3 may actually be protective against Aβ_1-42_-associated vSMC death in vitro [278] and APOE2 is associated with maximal Aβ clearance by CHO cells [280]. Interaction with HDL, as occurs physiologically, appears to increase the affinity of APOE for Aβ [281] and the association of APOE with lipids also increases binding to LRP1 [282]. The lipoprotein-APOE interaction likely aids in membrane anchoring [283]. However, Aβ binding decreases the affinity of lipid-free APOE for membrane lipoproteins, as the Aβ and lipoprotein binding sites appear to overlap and this results in decreased APOE interaction with the phospholipid layer [284]. Thus, in the physiological context of AD, it is plausible that elevated Aβ reduces APOE association with the vSMC membrane and binding to LRP1, and increased Aβ association with APOE4 in particular may cause a pathological decrease in amyloid clearance by vascular cells.

APOE4 heterozygosity is associated with increased CAA severity [285]. Sporadic CAA can be divided into two types, depending on the presence or absence of capillary Aβ deposition, with a strong association between APOE4-positive status and the appearance of capillary CAA [286,287]. Capillary CAA in AD patients is correlated with more severe parenchymal plaque pathology and molecular alterations in perivascular astrocytes, while the non-capillary CAA subtype does not appear to be associated with the APOE4 allele [287]. The APOE4 homozygous genotype in human AD is also associated with an increase in fibrinogen deposition in cortical vessels—in the lumen, vessel walls and medial layer, which could have implications for the weakening of vessel walls, disturbed hemodynamics and the development of CAA [288]. Fibrinogen can interact directly with Aβ deposits on the vasculature and fibrinogen clots associated with plaques are particularly resistant to fibrinolytic degradation, potentially leading to blood vessel occlusion and chronic inflammation around vessels [289,290,291]. Thus, the APOE4 association with fibrinogen accumulation could worsen vascular outcomes in AD. Indeed, high plasma fibrinogen levels appear to be associated with increased AD risk, due mostly to its hemostatic role and contribution to vascular pathology [292].

It is also worth noting that the saturated uptake of soluble Aβ species by human vSMCs appears to cause disruptions to smooth muscle actin networks and eventually leads to cell death in vitro [278,293,294]. Aβ binding to lipids in the cell membrane is also reportedly associated with cytotoxicity in vitro [294]. Cholesterol levels have a modifying impact on membrane-binding affinity, with higher cholesterol levels increasing Aβ cell membrane binding and increasing cell mortality [294]. This may also be worsened by APOE4-mediated Aβ degradation deficits, leading to the pathological accumulation of Aβ within vSMCs and other vascular cell types and contributing to increased cell mortality. It was also reported that vSMCs in vitro undergo degenerative changes in response to freshly solubilized Aβ_1-42_, but not to a pre-aggregated form [293,295]. This is in contrast to neurons, which are sensitive to pre-aggregated Aβ_1-42_ in vitro, suggesting that Aβ-activated cell death pathways may differ in vascular cells and neurons [295].

LRP1-associated dysregulation in perivascular brain pericytes may also contribute to AD vascular pathology in a similar manner as in other vascular cell types. As in endothelial cells and vSMCs, LRP1 receptors in pericytes have been shown to mediate Aβ internalization and clearance [243]. It has been shown that human brain pericytes acutely exposed to Aβ_1-40_ in vitro upregulate LRP1 and LDLR, possibly as a compensatory mechanism, resulting in enhanced Aβ uptake and precipitating cell death [274]. As discussed earlier, pericyte loss is well attested in human AD and in AD mouse models and likely contributes to neurovascular coupling deficits and progressively greater impairments in Aβ clearance capacity. Hippocampal NG2-positive pericyte loss is related to Aβ_1-40_ load in patients [184]. Fibrillar Aβ_1-40_ reduces pericyte proliferation and activates apoptotic cell death pathways in vitro [184]. On the other hand, monomeric Aβ_1-40_ was found to increase pericyte survival in vitro [184], suggesting a potentially protective effect. Laminin α5-positive pericytes, which are assumed to represent a mature quiescent phenotype, were shown to be unaffected in the AD hippocampus, which might suggest that pericyte subpopulations react differently to AD-related pathology [184]. A positive correlation was found between NG2-positive pericytes in the AD hippocampus and total Aβ_1-40_ in dissociated tissue, and considering the putative protective effect of monomeric Aβ_1-40_, this led the authors to speculate that this might suggest a pro-pericyte effect for monomeric Aβ_1-40_ [184]. It is interesting to note, as mentioned earlier, that another study found vSMCs to be selectively sensitive to monomeric Aβ_1-42_, while monomeric Aβ_1-40_ did not cause vSMC death in vitro [293]. This group also found that pre-aggregated Aβ_1-42_ did not cause vSMC mortality, unlike the monomeric form of Aβ_1-42_, which increased cell death [295]. The finding that pericytes, by contrast are sensitive to fibrillar Aβ_1-40_-induced degeneration and protected by monomeric Aβ_1-40_ could perhaps suggest the existence of different pathways for the action of aggregated and monomeric Aβ species in human brain pericytes and vSMCs, but this requires further investigation.

It has been suggested that metabolic processes within vascular cells in AD may contribute to the worsening of parenchymal Aβ deposition. As mentioned earlier in this review, the AD vasculature may be involved in the synthesis and release of several neurotoxic agents. In addition to causing vSMC degeneration, exposure to high levels of Aβ_1-42_ in vitro appears to precipitate an increase in APP expression and the synthesis of soluble Aβ in vSMCs [293]. This could conceivably contribute to Aβ deposition processes in the surrounding parenchyma while vascular cells themselves continue to undergo degeneration, suggesting the potential for a direct and reciprocal link between vascular and neuronal degeneration. However, this upregulation of Aβ synthesis appears to be mediated solely by the soluble rather than aggregated form of the protein [295]. Angiopathic blood vessels are anatomically associated with degenerating tau aggregate-containing neurites in the AD brain, similar to those associated with the cores of senile plaques [180,296] and this has been associated with the exudation of Aβ fibrils from these angiogenic vessels [195]. Amyloid plaques around vessels are also closely associated with networks of astrocytic processes [180]. Such amyloid fibril exudation from small blood vessels was noted in early electron microscopy studies and has been suggested to contribute to the formation of senile plaques [297,298]. In the Tg-SwDI mouse model of CAA, it was recently shown that amyloid “seeds” composed of a mutant CAA Aβ variant were capable of serving as scaffolds for the assembly of wild-type Aβ fibrils by cerebral capillaries, resulting in a purely vascular pattern of amyloid deposition [299]. It is unknown whether a similar mechanism could be at play in the AD brain. However, given recent interest in pathogenic Aβ seeding and the spread of amyloid pathology in the AD brain [300], this is an area that could provide some interesting insights into the involvement of the AD vasculature in the early generation and spread of amyloid pathology.

#### 2.4.4. The Role of Aβ in Aberrant Angiogenesis and BBB Dysfunction

Another interesting area is the potential link between amyloid deposition, aberrant angiogenesis and BBB disruption. Aged (18+ month-old) Tg2576 mice with the human APP_sw_ Swedish mutation exhibit increased angiogenesis, increased vascular density, abnormal morphology of tight junctions composed of occludin and tight junction protein-1 and compromised BBB function, but without significant vascular cell death [127]. Increased vessel density was also reported in the human AD hippocampus and cortex in the same study [127]. In vitro, chronic Aβ_1-42_ treatment of endothelial cells resulted in alterations in occludin, claudin-1, ZO-1 and ZO-2, proteins integral to endothelial tight junction structure and function [204,205], and the inhibition of RAGE or intracellular calcium influx rescued ZO-1 [205]. Tg2576 mice appear to display BBB changes at 4 months of age, prior to the development of plaques, suggesting a direct effect by soluble Aβ on the vasculature in these mice [301]. It is possible that abnormally increased angiogenesis and related changes in junction protein distribution contribute to BBB disruption in these AD models. The immunization of Tg2576 mice with Aβ has been shown to repair BBB permeability and immunization prior to AD symptom-onset appears to prevent aberrant angiogenesis and the impairment of BBB permeability [302,303]. Aβ vaccination has previously been shown to elicit reductions in plaque load by precipitating a humoral response and BBB protection might be achieved by a reduction in circulating Aβ. Aβ vaccination has been shown in past studies to achieve several positive outcomes in animal models but testing in humans was halted as a result of adverse effects in some participants [304].

On the other hand, others have shown an anti-angiogenic role for Aβ peptides. Aβ_1-40_ applied to human middle temporal artery and rat aorta explants in Matrigel causes a significant reduction in microvascular outgrowth [305]. A similar effect is observed in vitro, with reduced formation of capillary-like networks by human brain endothelial cells and in vivo, where inhibition of angiogenesis was seen in the chick embryo chorioallantoic membrane [305]. A study utilizing a mouse model with the Dutch Aβ_1-40_ mutation, a model of a hereditary CAA phenotype, appeared to show that both this mutant Aβ_1-40_ and wild-type Aβ_1-40_ cause impairments in angiogenesis by reducing the phosphorylation of fibroblast growth factor receptor-1 (FGFR-1); phosphorylation of the receptor is a necessary prerequisite for the binding of extracellular fibroblast growth factor-2 (FGF-2) and the subsequent endogenous synthesis of FGF-2, which plays a role in the maintenance of endothelial integrity [306]. However, pro- or anti-angiogenic effects in these studies may be the result of differing Aβ concentrations. Aβ_1-40_ was shown to enhance FGF-2-mediated angiogenesis at low concentrations, while higher concentrations caused the downregulation of FGF-2 synthesis, as well as greater endothelial cell apoptosis [306,307]. Interestingly, FGF-2 overexpressing cells appear to be resistant to Aβ_1-40_-mediated pathological processes in vitro [307] and the stimulation of FGF-2 synthesis in endothelial cells may thus be vasoprotective in AD and CAA. Soluble intermediates of Aβ aggregation have been shown to cause increased adhesion and transmigration of monocytes across the endothelial layer without causing endothelial death, while unaggregated Aβ had no effect on this process [308]. This adds to other reports that some small Aβ species like Aβ_25-35_ can, at low concentrations, increase endothelial permeability [309]. Both Aβ_25-35_ and Aβ_1-40_ induce apoptotic endothelial cell death pathways in vitro following prolonged exposure at high concentrations [309,310] and these cells are particularly apoptotic in the vicinity of Aβ_1-40_ deposits [309], which could be relevant to the pathological process in human AD. Indeed, as mentioned earlier, string vessels free of endothelial cells are more frequently observed in regions of the AD brain with high Aβ load [123]. There is in vitro evidence that soluble Aβ_1-40_ may be capable of crossing an endothelial monolayer by a diffusional process and at higher concentrations causes progressively greater impairments in barrier function, possibly through RAGE-Aβ interactions at the luminal membrane [311]. However, it is questionable whether Aβ diffusion across the BBB can occur in vivo, where transport across the BBB is highly regulated in several ways. Early aberrant amyloidogenesis and vascular Aβ aggregation could thus contribute to BBB dysfunction at an early stage in the disease, by precipitating hypervascularization, junction protein dysfunction and apoptotic cell death.

Aβ also plays a role in the increased infiltration of innate immune cells across the BBB, as previously described. In both the human AD brain and in AD mouse models, where neutrophil migration across the BBB is increased, neutrophils were found to be closely associated with Aβ plaques [216]. As previously stated, activated microglia also co-localize with vascular and parenchymal Aβ plaques [180,312] and this may be intended as a protective mechanism. However, as discussed, excessive accumulation and activation of immune cells may have detrimental effects and contribute to neurodegenerative changes. Soluble Aβ_1-42_ administration causes neutrophils to rapidly adhere to the endothelium through ICAM-1, mediated by lymphocyte function-associated antigen-1 (LFA-1) [216]. Monocyte adhesion to endothelial cells and migration across the endothelial monolayer has been shown to be stimulated by Aβ_1-40_ in vitro [313], mediated by endothelial RAGE and platelet endothelial cell adhesion molecule-1 (PECAM-1) [313]. As mentioned earlier, soluble aggregation intermediates of Aβ stimulate monocyte adhesion and transmigration, while unaggregated monomeric Aβ_1-40_ and Aβ_1-40_ fibrils do not appear to do so [308].

Thus, vascular Aβ clearance deficits contribute significantly to the development of CAA in AD and possibly play a role in parenchymal Aβ deposition. Furthermore, Aβ species in AD exert a direct effect on vascular cells, potentially contributing to cell mortality, changes in contractility, BBB permeabilization, immune cell transmigration and a variety of pathological changes at the molecular level.

### 2.5. Tau Pathology and Vascular Dysfunction

As with amyloid pathology, there is emerging evidence directly implicating pathological tau in AD vascular dysfunction. It was recently shown that tau oligomers are highly associated with endothelial cells and vSMCs in the cerebral vasculature in human AD, and in Tg2576 mice that display CAA, these were also found to be associated with cerebral amyloid deposits [314]. Tau appears to be far more significantly associated with CAA-affected arteries and arterioles than those without Aβ deposition [315]. Tau load in the AD brain has previously been shown to be associated with levels of Aβ_1-40_—the primary Aβ species in CAA plaques—but not Aβ_1-42_, and it was suggested that the p-tau pathology here is a consequence of microglial processing of excess Aβ_1-40_ [316]. This suggests that tau deposition in the cerebral vasculature in AD could contribute to vascular deficits and that there may be a link between Aβ accumulation in vessels and the later development of vascular tau pathology. Tau deposits are known to be cytotoxic [317] and could thus also contribute to vascular cell death. However, compared with Aβ, relatively little is known about the effect of tau or p-tau deposition in the AD vasculature and this represents an open area for future study.

In aged (15-month-old) Tg4510 mice, which overexpress the mutant P301L familial frontotemporal dementia tau protein, a variety of vascular abnormalities have been observed, including reduced vessel diameter, disturbed vessel architecture and an increase in atrophy-corrected blood vessel density in the cortex that declines after 15 months [318]. Interestingly, in one study, this was associated with the endothelial upregulation of several genes related to angiogenesis and some of these genes are also upregulated in endothelial cells from human AD cases (Braak stage V/VI) and from Tg21221 mice which overexpress a non-mutant human tau [318]. Plasminogen activator inhibitor-1 (PAI-1) is a serine protease inhibitor that is associated with angiogenic changes in endothelial cells. PAI-1 was found to be upregulated in human AD and in the mouse models of tauopathy utilized in this study [318]. The transient increase in vessel density in Tg4510 mice is interesting and similar transient changes in human AD might help to explain the inconsistencies between studies reporting increases or decreases in vascular density in various brain regions. However, caution must be applied in extending results from a non-AD mouse model with key pathological differences from the human condition.

### 2.6. Vascular Cells as Inflammatory Mediators in the AD Brain

Inflammation is ubiquitous in vulnerable areas of the AD brain, mediated by diverse cell types, and a wide range of pro-inflammatory chemical signals are upregulated in the disease. Microglia and astrocytes are the primary cellular mediators of inflammatory processes in the human brain. In addition to being activated by a wide variety of pro-inflammatory signals, activated microglia also secrete a range of these molecules themselves, including cytokines, chemokines, growth factors and complement proteins [319]. In AD, reactive astrocytes are also reported to release several pro-inflammatory agents [320]. The role of Aβ as an activator or potentiator of inflammation in the AD brain has been confirmed in numerous studies [321,322,323,324]. There is a growing appreciation for the sensitivity of cerebral vascular cells to inflammatory signals and the upregulated release of such signals by activated microglia and astrocytes no doubt has consequences for inflammatory activation in endothelial cells and pericytes. In this section, we will discuss how the inflammatory profile of the AD brain may precipitate changes in vascular function and the role that vascular cells play as participants in the inflammatory process in AD and as mediators of inflammation more generally.

In the AD brain, it has been shown that microvessels contain elevated levels of interleukin-1β (IL-1β), transforming growth factor-β (TGF-β) and macrophage inflammatory protein-1α (MIP-1α), and they also secrete much higher levels of interleukin-6 (IL-6) than control vessels [325,326]. In addition to this, the incubation of isolated AD vessels in serum-free media results in significantly increased release of IL-1β, interleukin-8 (IL-8), TGF-β, MIP-1α and tumour necrosis factor-α (TNF-α) compared with control cerebral blood vessels [160,325,326]. Human brain endothelial cells treated with TGF-β have been shown to increase their secretion of other pro-inflammatory signals like IL-1β and TNF-α [327], suggesting that vascular-derived inflammatory signals may act on the vasculature itself in an inflammatory feedback loop, precipitating greater vascular-related dysfunction in addition to deleterious effects on neurons and other cell types. TGF-β is involved in the regulation of several other processes in vascular cells, including angiogenesis, endothelial proliferation, vSMC differentiation, ECM deposition and interactions between vascular cells in the neurovascular unit [328], and this cytokine plays a role in both neurotoxic and neuroprotective processes. Thus, the implications of heightened vascular TGF-β are likely complex. One interesting role of TGF-β1, an isoform of TGF-β, is in the regulation of Aβ deposition and synthesis in the AD brain. In both aged wild-type (WT) mice and hAPP mice overexpressing astrocytic TGF-β1, there is more rapid deposition of Aβ along cerebral microvessels, which develop pathology reminiscent of CAA and AD [329]. Indeed, severe CAA in AD cortical tissue was found to be associated with strong TGF-β1 vascular immunoreactivity and TGF-β1 mRNA levels in the frontal cortex were correlated strongly with the extent of CAA [329]. TGF-β1 is significantly upregulated in the AD frontal cortex [329] and this could be the result of the inflammatory activation of multiple cell types including endothelial cells and perivascular astrocytes. Rats intravenously injected with Aβ_1-40_ in combination with bilateral injection of TGF-β1 into the thalamus display more significant amyloid pathology than rats injected with Aβ_1-40_ alone [330]. TGF-β1 has been shown to drive Aβ_1-40_ and Aβ_1-42_ synthesis in astrocytes, but not neurons, by inducing APP overexpression [331,332,333]. Whether the same holds true in endothelial cells remains to be seen. TGF-β1 also plays a role in ECM deposition [334] and decreased endothelial barrier integrity has been observed in vitro following the stimulation of human brain endothelial cells by TGF-β1 [335]. Strong vascular upregulation of TGF-β in AD could thus have important implications for amyloidogenesis and BBB remodeling, contributing to both neuronal and vascular pathology. IL-1β may also increase amyloidogenesis and has been shown to stimulate APP expression in astrocytes, neurons, human umbilical vein endothelial cells and mouse brain endothelial cells [332,336,337]. TNF-α has been shown to decrease barrier function in an in vitro BBB model and to cause significant upregulation of ICAM-1 in these cells [338,339]. TNF-α upregulation in the AD cerebral vasculature could thus contribute to the dysregulation of BBB stability in AD. TNF-α upregulation may also precipitate the reorganization of ECM networks and cause increases in BBB permeability [340]. The activation of endothelial cells by IL-1β, TNFα and lipopolysaccharide (LPS) in vitro brings about NFκB nuclear translocation and it has been shown that this causes the rapid loss of barrier function in the endothelial layer [335].

As described earlier, immune cell transmigration across the BBB is upregulated in AD. While this process may be mediated in part by impairments in BBB integrity, the disturbed vascular expression of chemokine proteins is likely to be involved. However, as with other inflammatory mediators, the impact of vascular chemokine dysregulation on neurodegeneration may be complex. MCP-1 plays an important role in the trans-endothelial migration of macrophages and monocytes into the brain [341]. MCP-1 upregulation has been demonstrated in cerebral microvessels isolated from AD brains [325] and in the AD mouse cerebral vasculature. CAP37 (Cationic antimicrobial protein Mr 37 kDa) is involved in the adherence and migration of monocytes and mononuclear cells at the endothelial surface, alongside a diversity of other functions in these cells. CAP37 has been reported to be expressed by endothelial cells in AD brain microvessels, but not in controls [342]. CAP37 expression was shown to be inducible in rat brain endothelial cells in vitro in response to Aβ_1-40_ and inflammatory mediators like TNF-α, interleukin-1α (IL-1α) and LPS [342]. In AD, the upregulation of these and other chemokine factors in endothelial cells may thus contribute to the increased recruitment of immune cells to the BBB and their transmigration into the brain parenchyma. Endothelial ICAM-1 mediates leukocyte and neutrophil adhesion to endothelial cells, facilitating transmigration across the BBB [343,344]. The endothelial induction of ICAM-1 by TNF-α and IL-1β [338,339] may thus have consequences for immune cell activation and entry into the AD brain. TNF-α also induces soluble VCAM-1 release by human cerebral endothelial cells in vitro, which is potentiated by interferon-β (IFN-β) [345]. ICAM-1 expression is regulated by NFκB, so the TNFα- and IL-1β-induced nuclear translocation of NFκB in human brain endothelial cells [335] may play a role in ICAM-1 induction. TNFα-mediated ICAM-1 induction involves the activation of the c-Jun N-terminal kinase (JNK) [346]. Thrombin is another mediator of leukocyte adhesion at the endothelium and has also been shown to increase ICAM-1 induction in human umbilical vein endothelial cells through JNK activation [347]. Thrombin can also increase ICAM-1 and VCAM-1 mRNA expression in human brain endothelial cells [348]. As mentioned previously, thrombin is upregulated in AD brain microvessels and senile plaques [160,349]. It has been shown that thrombin can activate brain endothelial cells in vitro, causing the upregulation of several CXC chemokines related to neutrophil chemotaxis, including CXCL1, CXCL2, IL-8 (CXCL8) and IP-10 (CXCL10) [348]. IL-8 was reported to be strongly upregulated in response to thrombin, perhaps consistent with the enhanced release of IL-8 by AD brain microvessels [325]. Thrombin can also induce MCP-1 mRNA expression in human brain endothelial cells [348]. RANTES (regulated on activation, normal T-cell expressed and secreted) is a chemokine expressed by endothelial cells and is a potent chemoattractant signal for a range of immune cell types, including monocytes, T cells, NK cells, dendritic cells, leukocytes and eosinophils [350]. RANTES is known to be upregulated in AD cerebral microvessels [351]. Interestingly, in vitro experiments with primary rat cortical neurons have demonstrated a neuroprotective effect for this chemokine, with RANTES treatment improving neuronal survival and also reducing thrombin- and sodium nitroprusside-induced neuronal death [351].

The induction of vascular inflammatory signalling by Aβ is known to occur in astrocytes and microglia in the AD brain, but a similar mechanism is now being revealed in cerebrovascular cells. In human aortic endothelial cells, Aβ_1-40_ treatment was shown to cause the upregulation of interferon-γ (IFN-γ) while Aβ_1-42_ induced the expression of IL-1β and IFN-γ [352]. In these cells, IFN-γ receptor (IFN-γR) expression was also increased with Aβ_1-42_ treatment, while Aβ_1-40_ had the same effect in human aortic smooth muscle, suggesting differential activation of these vascular cells by different Aβ species [352]. CD40 and IFN-γR upregulation in both endothelial and smooth muscle cells was enhanced by both Aβ species and also by IL-1β and IFN-γ directly. Moreover, CD40 upregulation has a positive feedback effect on Aβ-induced IL-1β and IFN-γ upregulation [352]. Taken together, Aβ may induce self-amplifying inflammatory cascades in endothelial and smooth muscle cells, contributing to more pronounced inflammatory outcomes. Cerebral endothelial cells in AD are now known to be activated by Aβ_1-40_ through a JNK-AP1 signalling pathway, causing the upregulated expression of diverse inflammatory mediators including MCP-1, IL-6, IL-8, IL-1β, CXCL1, CXCL2/macrophage inflammatory protein-2α (MIP-2α) and macrophage inflammatory protein-2β (MIP-2β) [353,354]. The involvement of JNK pathway activation could have consequences for the expression of other inflammatory genes regulated by this pathway like ICAM-1 [346]. In the AD brain, CAA is associated with the strong activation of infiltrating immune cells and microglia [209,355] and vascular cell activation could also occur in a similar manner.

Oxidative stress is a key pathological feature in the AD brain, related to perfusion deficits and several other factors. Indeed, several studies have demonstrated the interplay between oxidative stress and inflammation. The treatment of rat cerebral endothelial cells with H_2_O_2_ and the generation of reactive oxygen species by menadione treatment both result in the upregulation of MIP-1α release [326]. As stated earlier, human AD microvessels have been shown to upregulate MIP-1α expression and secretion [326] and hypoxia may be involved in the modulation of vascular inflammatory profiles in AD. Rat brain endothelial cells under hypoxic conditions upregulate several inflammatory gene mRNAs, including IL-6, thrombin, MCP-1 and MMP2, as well as HIF-1α [349]. HIF is one of the main transcription factors implicated in hypoxia-mediated adaptive changes in cells [356] and HIF-1α is overexpressed by the AD cerebral vasculature [160]. This could thus be a key mechanism by which inflammatory signals are modulated in the AD vasculature. Thrombin upregulation in AD microvessels may also impact on this system. It has been shown that rat brain endothelial cells respond to thrombin in vitro by upregulating HIF-1α expression [160]. Moreover, the inhibition of thrombin signaling in these cells, in combination with induced hypoxia, ameliorates hypoxia-induced inflammatory changes [349]. This implicates thrombin as an important regulator of vascular cell responses to hypoxia. Oxidative stress may itself be precipitated in the vasculature by increased cytokine stimulation and it has been shown that TNFα, IL-1 and IFN-γ treatment can cause the generation of reactive oxygen species in cultured endothelial cells, which seems to in turn stimulate the secretion of MCP-1 and IL-6 [357].

In recent years, pericytes have emerged as a major cellular mediator of neuroinflammation, responding to and secreting a wide array of pro- and anti-inflammatory signals. Although the role of pericytes in inflammation has not been well studied in the context of AD, it is likely that they are active in the process and sensitive to the widespread inflammatory changes in the surrounding milieu. Thus, it is worth discussing, in brief, the contribution of pericytes to inflammation in the brain. It has been demonstrated that primary human brain pericytes from meningeal explant cultures respond to pro-inflammatory cytokines like INF-γ, TNFα, IL-1β and LPS by significantly upregulating the expression and secretion of the chemokine inflammatory mediators IP-10 and MCP-1 [358]. Pericytes react to several pro-inflammatory stimuli with gene expression changes in key molecular systems related to neuroinflammation, including genes for interleukins, adhesion molecules and chemokines and this may involve the nuclear translocation of the NFκB transcription factor [187,358,359]. Pro-inflammatory signals have been shown to stimulate the upregulation of adhesion molecules like ICAM-1 and VCAM-1 and the release of the chemoattractant protein macrophage migration-inhibitory factor (MIF), which facilitates the recruitment of leukocytes to the vascular surface and their migration through the vessel wall into the brain parenchyma [359,360]. The release of IL-8 by activated pericytes may promote neutrophil recruitment and migration across the vessel wall and this is further facilitated by MMP-9 secretion and ICAM-1 expression [361,362,363]. TNFα stimulation of pericytes, in particular, is linked to increased microglial activation mediated by MMP-9, MIP-1 and IL-6 [364]. T-lymphocyte activation is also facilitated by VCAM-1 and ICAM-1 upregulation [365,366]. The over-induction of inducible nitric oxide synthase (iNOS) by pro-inflammatory cytokines may also contribute to downstream inflammatory processes and the resulting nitric oxide (NO) generation may contribute to local free radical damage [367]. Pericytes thus play a dynamic role in inflammatory mediation and a better understanding of their contribution to inflammation in AD may provide deeper insights into the vascular contribution to this aspect of disease pathology.

## 3. Systemic Vascular Health and Dementia Risk

It has long been suspected that there is a connection between systemic vascular health and AD risk. Following several early reports of vascular pathology in AD, as already described in this review, many epidemiological studies have sought to determine whether AD can be linked to more general cardiovascular impairment and whether treating patients for cardiovascular disease has an impact on the development or progression of AD [368,369,370,371,372]. Some of the commonly investigated cardiovascular risk factors include hypertension, atherosclerosis and hypercholesterolemia [373,374,375]. And underlying all these risk factors is APOE genotype, which appears to interact with systemic vascular risk factors in a complex manner [25,28,369].

### 3.1. Hypertension

Hypertension has been extensively studied as a risk factor for the development of AD [369,371,376]. Hypertension is of particular interest as a risk factor due to its high prevalence - approximately one-third of adults worldwide are affected by hypertension [377]. Studies have typically focused on either the prevalence of concurrent AD and hypertension or whether mid-life hypertension can be predictive of later development of AD [369,376].

Epidemiological studies have produced conflicting results regarding the relationship between hypertension and AD [376,378]. Studies investigating AD risk based on mid-life blood pressure have found positive associations between hypertension and AD, whereas cross-sectional studies show an association between low blood pressure and concurrent AD [369,378]. It has been suggested that low blood pressure in AD patients is secondary to cortical atrophy [370]. For this reason, it is likely that mid-life blood pressure findings are more predictive of AD than low blood pressure at an advanced age.

Studies which have been carried out to assess the impact of anti-hypertensive drugs on the development of AD have yielded promising results. It has been shown in Sprague Dawley rats that angiotensin II may play a role in the accumulation of Aβ [379] and so treatments which downregulate the angiotensin axis may play a double role in the treatment of AD, preventing both amyloid accumulation and damage to small vessels [380,381].

A post-mortem study found that patients who had been treated with angiotensin receptor blockers (ARBs) were less likely to receive a diagnosis of AD compared with those who were treated with other classes of antihypertensives or those not treated with antihypertensives and this association held true even after controlling for APOE genotype [382]. A recent meta-review of studies in the field concluded that ARBs were protective against the development of AD in animal models and human observational models and in randomized control trials (RCTs) [371]. A recent RCT found that treatment with a combination of an ARB and a diuretic succeeded in lowering blood pressure but had no significant effect on cognitive decline over the duration of the study, a mean period of 5.7 years [383]. A possible reason for this is that the study participants were over the age of 70 and by this time, the impairment may have been too great. This is consistent with other findings, detailed above, indicating that blood pressure at mid-life is a better predictor of cognitive decline than blood pressure in old age.

Centrally acting angiotensin-converting enzyme (ACE) inhibitors are associated with reduced rates of cognitive decline in patients with established AD [24,384,385], independent of blood pressure reduction [24]. However, this association may not persist in the long-term [385] and is not seen in carriers of APOE4 [24]. It should also be noted that one study showed an increase in mortality for patients with AD who were treated with ACE inhibitors compared with those who took ARBs [372], although it is uncertain whether this represented an interaction with AD vascular pathology.

### 3.2. Atherosclerosis

The evidence for the role of atherosclerosis in the development of AD is mixed. Studies have found an association between AD and atherosclerosis at autopsy [25,386,387], and between intracranial atherosclerotic disease and mild cognitive impairment (MCI) and dementia [388]. Others, however, have shown only an association with dementia and MCI generally and not with AD specifically [389]. APOE genotype may play a role in determining AD risk in individuals with atherosclerosis, as the presence of the APOE4 allele appears to strengthen the association with AD [25].

APOE4 status is also associated with risk of atherosclerosis [390,391], though the mechanisms underlying this relationship are not fully understood. As in AD, APOE4 increases the likelihood of disease, while APOE2 has been found to be protective [391,392].

Sex differences may also play a role in defining the strength of the association between atherosclerosis and AD. It has been found that the increase in risk of atherosclerosis conferred by the APOE4 allele is greater in women than in men [393]. For example, in the Framingham study, an association between carotid atherosclerosis and APOE genotype was reported in women, whereas in men, this association was only seen in the presence of diabetes [394]. Sex differences are also important in the development of AD [395,396]. Women are at higher risk of developing AD than men [397,398] and, as with atherosclerosis, it has also been found that the presence of at least one APOE4 allele is associated with a greater increase in AD risk for women than for men [393,399]. 

These factors complicate the relationship between atherosclerosis and AD, and differences in patient selection and whether studies controlled for APOE genotype may go some way towards explaining why results in this area have been mixed. Currently, it is unclear whether there is an independent relationship between atherosclerosis and AD or whether this is primarily due to their shared association with APOE genotype.

### 3.3. Hypercholesterolemia 

Because of its links to CVD and APOE genotype [31,391], hypercholesterolemia has been suspected by many to be involved in the development of AD, but research so far has not yielded convincing results. While associations have been found between vascular dementia and blood lipid levels, the same has not been demonstrated for AD [400,401]. Additionally, treatment with lipid-lowering drugs has been found not to influence AD risk [401,402]. Cross-sectional studies have found that high cholesterol is associated with some features of AD such as neuritic plaques, but not NFT pathology [403]. Based on these findings, at this time, there is insufficient evidence in support of cholesterol-lowering drugs as a preventative treatment for AD.

## 4. Alzheimer’s Disease and Vascular Dementia

AD represents the most prevalent form of dementia worldwide. However, it is important to keep in mind that the disorder exists on a spectrum of dementias, many of which present similarly and exhibit similar patterns of progression. Particularly in prodromal patients or patients with MCI, it is not possible to ascertain whether progression to AD will occur or if the cognitive impairment is indicative of another form of dementia—this is undoubtedly a major limitation in studies investigating perfusion deficits and other changes in very early AD.

Vascular dementia (VD) is a term describing a heterogeneous family of dementias involving cognitive deficits stemming from or related to impaired brain blood flow [404]. However, as described in this review, cardiovascular and neurovascular abnormalities, including changes in perfusion, are well attested features of AD. Further complicating matters is the observation that as many as one-third of patients with VD go on to develop pathological signs of AD including amyloid plaques and NFTs [405], perhaps consistent with the idea that AD may stem in part from cerebrovascular insults or dysfunction. However, AD lesions appear to be more common in VD patients with a lower volume of macroinfarction, suggesting that AD pathology may be more related to microinfarction rather than to larger-volume macroinfarction [405]. Cerebrovascular lesions are also commonly observed in patients with AD [406] and AD patients are at heightened risk for stroke and intracerebral hemorrhage [407]. The term “mixed vascular-Alzheimer dementia” (MVAD) has been used to describe the appearance of neurodegenerative AD dementia alongside cerebrovascular disease. Some criteria for the diagnosis of MVAD have been suggested, despite the difficulty in clinically differentiating MVAD from AD in many cases and the lack of broad consensus on the matter [18]. Similarly, diagnostic guidelines for VD are unclear and the clinical features of the disease are very dependent on the specific vascular pathology and resulting neural implications [404,408]. Vascular dementia is sometime assessed by the observation of a temporal relationship between stroke and dementia onset, the detection of cerebrovascular lesions by neuroimaging and the exclusion of signs of other dementias like AD [408]. It has been suggested that MVAD be diagnosed by the presence of multiple vascular lesions or infarcts in the cortex, basal ganglia, thalamus, hippocampus and white matter, with an infarcted volume of 30–50 mL [409]. Differences in cognitive decline in specific domains have been suggested for patients with AD and subcortical VD [410] but other studies have found the evolution of cognitive decline to be so similar in AD and VD as to greatly limit its diagnostic potential [411].

Considering the significant symptomological overlap between AD and VD [412], the similarities in evolution of cognitive dysfunction [411], the potential causative relationship between cerebrovascular dysfunction and AD and the high prevalence of vascular lesions in diagnosed AD patients, many of the patients in the clinical studies covered earlier in this review would likely have been classifiable as MVAD patients and some of the prodromal patients with perfusion deficits and MCI could well have progressed to a more pure form of VD later in the course of the disease. While it wise to be cautious when considering MCI and prodromal cases as being a prelude to AD, it is likely that cases defined as “pure” AD or “pure” VD are simply extremes of a single neurodegenerative-vascular disorder, consistent with the vascular theory of AD [413]. Indeed, at autopsy, 60–90% of diagnosed AD patients show cerebrovascular pathology, with 30% showing signs of cerebral infarction [413]. A potentially causative link between vascular dysfunction and key signs of AD like amyloid deposition and NFT formation (as discussed earlier in this review) and the development of such AD pathologies in cases of advanced VD might indicate that the two conditions need not necessarily be separated while considering early AD pathogenesis and disease prevention. AD and VD patients appear to respond similarly to several drug therapies, including acetylcholinesterase inhibitors (AChEIs) and memantine [18]. A differentiation between AD and VD is perhaps more important in designing and tailoring therapies to patients who may occupy diverse points on the MVAD spectrum. It is possible that some patients would benefit more from therapies targeting vascular dysfunction, while others may benefit more from therapies targeting classic AD pathology. The differentiation of cases with more AD-like or more vascular pathology may also be important in clinical trials assessing therapies that target specific pathological processes.

## 5. Implications for Therapy and Drug Design

Over the last few decades, there has been a major push for the development of disease-modifying drugs targeting pathways related to Aβ and p-tau generation, aggregation and clearance, in accordance with the prevailing amyloid and tau hypotheses of AD. In addition, drugs targeting components of the cholinergic and glutamatergic pathways have been approved for symptomatic treatment and agents targeting several inflammatory microglial activation pathways are being investigated. However, a true preventative or curative therapy for AD remains elusive and it is becoming increasingly clear that a combinatorial approach to treatment is necessary, especially given our growing appreciation for the complex etiology of the disorder. As yet, there have been few published attempts to therapeutically target AD-associated vascular dysfunction, despite this being an early feature of AD that significantly influences disease outcome. However, several potential targets and mechanisms have been identified that may help to inform the design of novel vascular-targeted therapies for AD (Table 1).

Despite growing evidence for the contribution of BBB disruption to AD pathogenesis, little research has been conducted into the potential of vascular cells as drug targets for AD treatment. Several pathological processes in the AD vasculature present themselves as targets, including BBB breakdown and hyperpermeabilization, dysregulated immune cell migration into the brain, vascular inflammatory responses, vascular amyloidogenesis, aberrant angiogenesis, mechanisms of pericyte and endothelial loss and the release of neurotoxic compounds by the vasculature. In the previous section, we have made brief mention of therapies targeting systemic vascular risk factors like hypertension and hypercholesterolemia and their effect on AD risk as revealed by epidemiological studies. Here, we will focus specifically on aspects of AD neurovascular dysfunction that may yield promising results when considered as therapeutic targets.

As discussed earlier, cholinergic dysfunction is believed to be one of the key drivers of perfusion changes in the AD brain and there is a significant loss of cholinergic innervation to the cerebral cortex and hippocampus [103,104,414,415]. As a potent vasodilator, reduced ACh release at the vasculature results in initially focal and then more widespread increases in vasoconstriction [105]. The cholinergic hypothesis has been the basis for the development and trialing of several therapies for AD over the decades, in particular AChEIs like tacrine, galantamine, donepezil, rivastigmine and metrifonate, in the hopes of ameliorating the cholinergic deficit in the AD brain [416]. These drugs have been associated with the slowing of cognitive deterioration, but effects are quite heterogeneous. Donepezil, a common AChEI in the treatment of AD, has been shown to aid in the preservation of blood flow in several vulnerable brain regions in mild-to-moderate AD, following one year of treatment [417]. In a SPECT follow-up study of mild-to-moderate AD patients undergoing long-term AChEI therapy, it was observed that patients who saw clinical benefit from AChEI treatment exhibited stabilized perfusion profiles, whereas those who were resistant to AChEI therapy displayed perfusion deficits characteristic of AD [418]. This would seem to suggest that the preservation of cholinergic transmission in some patients has positive outcomes for perfusion and this may potentially contribute to improved outcomes. Rivastigmine was also reported to improve CBF in some brain regions in AD patients, in particular in the temporal cortex [419]. Recently, AChEIs were also shown to improve frontal lobe perfusion in AD patients following a year of treatment [420]. A study of the AChEI velnacrine revealed that a single dose was sufficient to improve regional perfusion and metabolism in AD patients and this was associated with slightly improved word recall [421]. Thus, the cognitive benefits provided by AChEI treatment in AD may be partially mediated by improved cerebral perfusion. Of course, AChEIs are not disease-modifying, as the loss of cholinergic innervation eventually becomes too great to compensate for. However, it should be noted that AChEIs are often prescribed following the clinical manifestation of the disorder, when pathology may be advanced. As discussed earlier, perfusion deficits may begin years to decades before the onset of clinical symptoms and the deleterious effects of these changes may guide the brain in a pathological direction. In addition, predisposing factors like family history of AD and APOE genotype may cause measurable changes in perfusion prior to the development of AD. The preservation of CBF and metabolism in the pre-AD brain may be of benefit in the prevention of metabolic and other deficits associated with reduced perfusion. Screening for AD risk factors in combination with diagnostic imaging and therapies to modulate CBF may provide some benefit at this stage, but this requires confirmation. In reality, such an early therapeutic intervention would depend on reliable screening techniques and the prescription of drugs to otherwise healthy individuals, which is impractical in the absence of factors associated with high individual risk. It is currently unclear whether very early interventions to prevent vascular perfusion deficits would be disease-modifying, and if not, whether they would provide any benefit in slowing symptomatic onset.

The therapeutic targeting of defective Aβ clearance mechanisms at the BBB may serve the dual purpose of reducing parenchymal Aβ load while reducing Aβ-associated pathology at the BBB including CAA and Aβ-mediated vascular inflammation. LRP1 is one of the most important mediators of Aβ clearance across the BBB. As discussed, LRP1 expression is significantly affected in the AD cerebral vasculature – in endothelial cells, vSMCs and pericytes. LRP1 expression also declines with age [422] and as described, APOE genotype plays a major role in the heterogeneities associated with LRP1 deficits in the AD vasculature. Thus, the rescue of LRP1 may potentially represent a novel therapeutic strategy in preventing Aβ build-up and deposition in early AD or in at-risk individuals. Alternatively, the inhibition of RAGE on endothelial cells may reduce the association of Aβ with endothelial cells and the entry of Aβ into the brain. It is currently unknown whether LRP1 augmentation is possible in the human cerebral vasculature. Pharmacological manipulation may involve the use of statins, which appear to increase LRP1 expression and amyloid clearance in in vitro BBB models [423] and to upregulate hepatic LRP1 in rats [424]. There is some evidence that statins may have beneficial effects in terms of lowering AD risk and improving cognition [425]. However, several clinical trials have failed to demonstrate efficacy and this is thus a controversial area in AD therapeutics [426]. Still, more research is required into the specific effects of statins on vascular LRP1 expression in vivo. LRP1 upregulation could also be a viable goal for gene therapy [427] and this is an area that is currently unexplored. Finally, as mentioned earlier, it has been shown that sLRP is also reduced in AD patients, potentially contributing to an increase in plasma Aβ and greater Aβ uptake into the brain. APP^+/sw^ mice treated with recombinant LRP1 ligand-binding domain IV (LRP-IV) displayed a significant reduction in cortical and hippocampal Aβ load, both vascular and parenchymal, as well as free Aβ [256]. It has thus been suggested that sLRP supplementation or replacement may reduce the BBB uptake of Aβ through endothelial RAGE, which is significantly upregulated in the AD microvasculature. Similarly, soluble RAGE (sRAGE) may also serve a similar function. RAGE inhibitors have reached clinical trial and have been tested in animal models, but the results of these studies have been mixed regarding efficacy and safety [428].

Another approach could involve the modulation of enzymatic amyloid degradation pathways in the AD brain. Neprilysin, a key enzyme in brain Aβ degradation pathways, has received considerable interest in recent years as a regulator of brain Aβ load [429]. Neprilysin is downregulated in the AD cortex [430] and in the cerebral vasculature in particular, likely contributing to deficits in amyloid degradation within vSMCs [263]. Interestingly, the downregulation of neprilysin in the AD brain appears to begin at an early stage. Neprilysin activity is reduced in the blood serum of patients with MCI [431] and neprilysin levels were reported to decline in the CSF of patients with early AD and amnestic MCI progressing to AD [432]. Neprilysin polymorphisms have been associated with the appearance of CAA and are correlated with its severity [433]. Thus, this could represent a target for intervention in the prevention or reversal of Aβ pathology in early AD. Genetic modulation of neprilysin expression may be a viable route to achieve this. It has been demonstrated in neprilysin-deficient SH-SY5Y neuroblastoma cells that histone deacetylase (HDAC) inhibitors like valproic acid can induce the transcriptional upregulation of neprilysin [434] and valproic acid can increase neprilysin expression and reduce plaque load in APP/PS1 mice [435]. Although HDAC inhibitors would not be selective for cell type, they could aid in the preservation of neprilysin activity in vascular cells. However, it is important to note that HDACs are involved in the transcriptional regulation of a large number of other important genes. Future research could help to identify more specific regulators of neprilysin expression. Other pharmacological approaches may also be useful in upregulating neprilysin expression in cerebral vascular cells, but current approaches are not targeted towards the vasculature. For example, somatostatin receptor 4 (SSTR-4) agonists have been shown to upregulate neprilysin expression in APP/PS1 mice and reduce soluble Aβ_1-42_ load [436]. Brain endothelial cells express all five subtypes of SSTRs and the vascular targeting of these receptors might be an option. Several other neprilysin-upregulating pharmacological treatments have shown similar effects on Aβ deposition in AD mouse models and other enzymes involved in amyloid degradation may potentially be targeted in a similar manner [437].

It has also been suggested that some pro-angiogenic treatments may be of benefit to AD vascular dysfunction, given the vessel degeneration that has been reported in the AD cortex. Previously, we discussed how VEGF expression is downregulated in the AD cortex and the pathological consequences of this. VEGF supplementation has been suggested as a way to rescue vascular deficits associated with impaired vascular proliferation. AD transgenic mice treated with VEGF display reductions in Aβ-induced endothelial cell apoptosis, stimulated endothelial proliferation, improved cognitive performance and reduced Aβ and p-tau load [152,153,154]. This may point to VEGF supplementation as a route by which vascular and other deficits may be treated in AD. Aβ-induced angiogenesis has been reported previously, as described earlier in this review. Given the role of aberrant angiogenesis in the remodeling of the vascular system and weakening of the BBB, as well as its likely contribution to perfusion deficits, strategies that prevent angiogenesis may theoretically have therapeutic benefit in AD. As described earlier, Aβ immunization in the Tg2576 AD mouse model has been shown to dramatically reduce hypervascularity and halt aberrant neoangiogenesis, likely through the abrogation of Aβ–mediated angiogenic pathways [303]. Indeed, Aβ immunization dramatically reduced Aβ plaque load, reduced the occurrence of activated microglia and improved vascular barrier function [302]. Thus, therapies that aim to directly reduce amyloid load in the AD brain may also be useful in the prevention of angiogenesis-related BBB deficits, as well as other vascular pathologies like CAA, Aβ-mediated vascular cell death and Aβ-mediated vasoactive effects. Several early Aβ immunization trials in AD patients were halted due to severe side-effects in some patients, including meningoencephalitis and intracerebral hemorrhage, but there is still interest in this therapeutic route and there are currently several such therapies in development and in clinical trials [438].

Aβ immunotherapy with anti-Aβ monoclonal antibodies (mABs) has emerged as a promising strategy in the reduction of brain Aβ levels without the severe side effects associated with Aβ vaccines—a few such anti-Aβ mAB immunotherapies are in clinical trial [439]. However, several recent trials have been discontinued after failing to meet primary endpoints. A murine homologue of aducanumab, a fully human anti-Aβ mAB, was shown to bind and reduce both soluble oligomeric and insoluble fibrillary forms of Aβ in the brains of Tg2576 transgenic mice [440]. A preliminary study with human aducanumab indicated that the drug was able to reduce fibrillary Aβ load in prodromal and mild AD patients when administered intravenously for one year (once per month) and that this reduction was dose- and time-dependent [440]. Considering these results, as well as the promising safety and tolerability profile of the drug in human AD patients [440,441], it was subsequently advanced to clinical trial. However, aducanumab failed to demonstrate clinical benefit in phase 3 trials and these trials were thus halted by Biogen and Eisai in early 2019. Crenezumab—a humanized anti-Aβ mAB with broader specificity for monomeric, oligomeric and fibrillary Aβ species—was not shown to significantly reduce amyloid burden or improve cognitive performance in a cohort of mild-to-moderate AD patients over 68 weeks, either at low (300 mg subcutaneous) or high (15 mg/kg intravenous) doses [442]. However, non-significant trends towards slowed plaque formation and reduced cognitive decline were observed in the high-dose patient group compared with controls, with post-hoc analysis indicating reductions in cognitive decline (ADAS-Cog12) particularly in patients with very mild AD (MMSE score of 22-26) treated with the high dose [443]. Crenezumab CSF levels were correlated with a significant increase in Aβ_1-42_ CSF levels [443], indicating that the antibody may indeed promote Aβ_1-42_ clearance. This drug progressed to phase 3 trials to test whether even higher doses (60 mg/kg, once per month) could potentially improve efficacy [442,443], but these trials were halted by Roche in early 2019 following the finding in an interim analysis that the drug was not likely to reach its primary endpoint. The drug is still however being tested for the slowing of cognitive decline in autosomal-dominant familial AD and this trial involves the administration of the drug to healthy individuals with the PSEN1 A280 familial AD mutation [444]. Regarding anti-Aβ mAB-associated changes in CSF Aβ levels, caution should be exercised in the interpretation of such CSF biomarker changes. Increased CSF Aβ_1-42_ and Aβ_1-40_ levels have been noted in patients treated with solanezumab, another humanized anti-Aβ mAB; however, this does not appear to be associated with significant reductions in brain Aβ in mild-to-moderate AD patients [445,446]. This drug also failed to demonstrate significant benefit in phase 3 trials in mild AD patients, although a non-significant slowing in cognitive decline was noted [446]. This could indicate that significant increases in CSF Aβ levels are not representative of plaque reduction or that plaque reduction would be associated with even higher CSF Aβ levels than those noted in these studies. Another two anti-Aβ mABs are currently in clinical trials—BAN2401, selective for Aβ protofibrils [447,448] and gantenerumab, selective for a specific folded conformation of aggregated Aβ [449]. Gantenerumab has thus far not met primary end-points in phase 2 trials but high-dose trials are in progress on the basis of post hoc results showing non-significant benefits in “rapidly-progressing” prodromal patients and patients with elevated Aβ CSF levels [450].

The failure, thus far, of anti-Aβ monoclonal antibodies in meeting clinical trial efficacy endpoints has led some to suggest that amyloid pathology is simply too advanced by the “mild AD” stage for these treatments to be effective. Interventions targeting Aβ accumulation may thus be more effective at an even earlier stage of disease progression [451,452]. A key consideration in drug design is the presence of both aggregated and monomeric Aβ species in the AD brain. While aggregated Aβ species are considered to be the primary mediators of AD neurodegeneration, Aβ monomers may also be involved in disease pathogenesis [453]. Aducanumab and other antibodies that exclusively target aggregated Aβ may not slow monomeric Aβ-induced pathological processes but whether this has any relevance to AD therapy is questionable. The specificity of anti-Aβ mABs for specific segments of the Aβ sequence may also preclude the clearance of smaller Aβ fragments that have been shown to exhibit neurotoxic effects in vitro and in vivo [454,455]. Drug delivery is another important issue, as delivery of mABs into the brain is greatly hindered by the BBB [456]. Many studies have noted that high mAB doses are associated with greater efficacy [439] and this may be in part due to the very low efficiency of drug delivery into the brain parenchyma. The targeting of vascular Aβ clearance pathways with sLRP or sRAGE supplementation, RAGE inhibition or through genetic LRP-1 upregulation may potentially promote vascular Aβ clearance without the design and delivery constraints associated with mABs. Indeed, a combinatorial approach could be of value here. The targeting of circulating monomeric Aβ by mABs may tip the equilibrium for vascular Aβ transport in favour of efflux [457] and modulation of vascular Aβ transport mechanisms may enhance this effect. Solanezumab was shown to increase monomeric Aβ clearance without binding plaques or oligomers and such mABs could be useful in this approach. Furthermore, mABs that increase plaque clearance may also mobilize soluble neurotoxic Aβ oligomers and in doing so also activate microglial-mediated inflammatory pathways and enhance vascular deposition of Aβ [457]; this could perhaps be ameliorated to some extent by modulation of vascular Aβ transport mechanisms. Although anti-Aβ monoclonal antibodies have been found to be well-tolerated by AD patients in general, studies have noted an increase in so-called amyloid-related imaging abnormalities (ARIA) at high doses, particularly in APOE4 carriers. While the precise causes of ARIA are unknown, this phenomenon is potentially related to the effects of mABs on Aβ mobilization and vascular deposition. A trial with aducanumab revealed an incidence of 41% amongst patients receiving a 10 mg/kg dose and the incidence of ARIA was even higher in APOE4-carriers [440]. This study also noted superficial siderosis in 9% of patients in the same dosing group, potentially indicative of micro-hemorrhage and increased edema [440]. Considering the high doses being administered in current clinical trials and the known association between mAB dose and ARIA, this is a potential concern for the development of these drugs. A combinatorial approach utilizing drugs that promote vascular Aβ clearance may thus be a valid direction for future inquiry.

Given the pro-inflammatory state prevailing in many parts of the AD brain and the widespread pathological dysregulation of inflammatory regulation and responses, neuroinflammatory processes are gaining importance as a focus of target identification. Much attention has been paid to inflammatory processes in glial cells, in particular microglia and astrocytes, as these have long been considered the key cellular mediators of neuroinflammation. However, vascular cells like endothelial cells and pericytes likely contribute to the inflammatory state of the AD brain and are themselves very sensitive to it. Targeting inflammatory processes in the vasculature may aid not just in the prevention of inflammation-mediated neuronal death but also in the preservation of BBB integrity, the modulation of immune cell entry into the brain, the prevention of vascular-mediated neurotoxicity and the rescue of cerebrovascular cells. Sunitinib, an inhibitor of vascular activation has recently been tested in transgenic AD mouse models. This drug has inhibitory activity against a range of receptor tyrosine kinases, including some that are highly associated with the vasculature and vascular function, like platelet-derived growth factor receptors (PDGFRs) and VEGF [458]. Sunitinib was confirmed to reduce significantly the expression of several pro-inflammatory molecules in transgenic AD mice, including TNF-α, thrombin, IL-6 and IL-1β, as well as the vascular expression of Aβ. Treatment with sunitinib improved cognitive performance in these mice and was found to reduce endothelial cell death in response to oxidative damage in vitro [458]. Thus, the specific targeting of vascular inflammatory activation may be a promising future avenue for ameliorating vascular dysfunction in AD. There is a need for more studies investigating these mechanisms in animal models of the disease and other drugs targeting vascular inflammatory pathways need to be tested. The general modulation of inflammatory state in the AD brain may also have a beneficial effect on vascular activation and there are several drugs at various stages of animal and clinical trials [459]. It is worth noting that non-steroidal anti-inflammatory drugs (NSAIDs) have long been believed to reduce AD risk, but these drugs have not shown efficacy in more recent AD clinical trials [460].

Some studies suggest that modulation of APOE levels might be beneficial in reducing amyloid levels, preventing tau-mediated toxicity and improving synaptic function [461,462], and this approach might be promising as a preventive therapy. However, bexarotene, a drug that has been shown successfully to increase brain APOE concentrations, thus reducing Aβ levels and reversing cognitive deficits in APP/PS1 mice [463], did not meet the expected outcomes in clinical trials. The drug was able to lower brain Aβ levels, but in APOE4 non-carriers only, and also caused an increase in serum triglycerides, which might increase cardiovascular risk [464]. Even so, there is a growing appreciation for the fact that APOE4 genotype determines responsiveness to and tolerance for certain pharmacological interventions, making this an important consideration in the design of therapies and in the genotype-specific tailoring of drug treatment regimens for AD. This is perhaps unsurprisingly, given the involvement of APOE in so many different mechanisms purported to underlie vascular pathology and disease risk. For instance, APOE4 genotype has been shown to be crucial in determining patient outcomes to some Aβ immunotherapies. Bapineuzumab is a humanized mouse monoclonal antibody against Aβ, believed to induce the BBB-mediated clearance of Aβ and uptake by microglia in a dose-dependent manner [465,466]. In human trials, bapineuzumab was found to produce potential treatment benefits based on the Disability Assessment for Dementia (DAD) scale and was relatively well tolerated in APOE4 non-carriers with very mild AD (MMSE threshold ≥ 20) [466]. APOE4 carriers, on the other hand, did not benefit from treatment and were at additional risk for vasogenic cerebral edema [466,467]. However, it was noted that CSF p-tau levels were reduced in APOE4 carriers with mild-to-moderate AD, but not in non-carriers [466]. There is growing evidence that menopausal decreases in estrogen levels are a major underlying factor for the heightened AD risk in women [468,469,470]. Estrogen replacement trials have produced conflicting results, but the APOE genotype has been demonstrated to differentially modulate the effects of estrogen therapy in AD [471]. The interaction between APOE and estrogen replacement is most likely multifactorial but includes effects on the cardiovascular system and Aβ accumulation [472]. Taking into account the differences in treatment outcome and side-effect profiles observed in APOE4 carriers versus APOE4 non-carriers, it is clear that this is a potentially important consideration in the development of efficacious and safe drugs for the treatment of AD [473].

## 6. Concluding Remarks

In this review, we have provided a detailed evaluation of perfusion and metabolic deficits in the AD brain and the contribution of diverse modes of vascular dysfunction to the pathological process in AD. As stated in the introduction, the degradation of the vasculature has long been neglected as an area of primary focus in AD research, with the assumption being that vascular abnormalities were explained by pathological processes elsewhere in the AD brain. However, decades of research clearly underscore the contribution of vascular factors at multiple stages of disease pathogenesis and the involvement of vascular risk factors preceding the onset of classical symptomatology and gross pathological changes. Existing theories of AD pathogenesis have been insufficient on their own in informing the design of disease-modifying pharmaceutical therapies and other avenues must be explored. Based on the findings presented here, it seems clear that vascular-mediated processes present a large, diverse and relatively unexplored set of therapeutic targets for the treatment of AD. Given the multimodal and global nature of the molecular, physiological and anatomical pathologies reported in the AD brain, it is becoming increasingly clear that future therapeutic design should be focused on the development of combinatorial approaches targeting multiple aspects of the disease mechanism. The treatment of vascular dysfunction and inflammation at an early stage in AD pathogenesis, or even in pre-symptomatic individuals, may greatly improve short and longer-term outcomes and perhaps ameliorate the pathological process itself.

## Figures and Tables

**Table 1:** The therapeutic targeting of vascular dysfunction in Alzheimer’s disease (AD)—some potential targets and strategies.

AD Pathology	Therapeutic Strategies	Potential Targets and Mechanisms
Aβ aggregation/clearance deficits	Targeting amyloid-β (Aβ) degrading enzymes	Neprilysin upregulation through histone deacetylase inhibitors like valproic acid. Neprilysin upregulation through vascular somatostatin receptor-4 (SSTR-4) stimulation.
	Targeting vascular Aβ transport processes	Soluble low-density lipoprotein receptor-related protein (sLRP) supplementation or replacement. Potentially with recombinant LRP1 ligand-binding domain IV (LRP-IV). Low density lipoprotein receptor-related protein-1 (LRP1) upregulation through gene therapy. Soluble receptor for advanced glycation end-products (sRAGE) supplementation. Receptor for advanced glycation end-products (RAGE) inhibition.
	Reducing total brain amyloid load	Aβ immunization. Aβ immunotherapy, for example monoclonal antibody-based therapies. BAN2401 and gantenerumab currently in clinical trials. Apolipoprotein (APOE) modulation, for example increasing brain APOE levels with bexarotene in APOE4 non-carriers.
Aberrant angiogenesis	Pro-/anti-angiogenic treatments	Vascular endothelial growth factor (VEGF) supplementation.
Vascular-mediated inflammation	Anti-inflammatory agents	Inhibitors of vascular activation, for example sunitinib. Non-steroidal anti-inflammatory drugs (NSAIDs). VEGF supplementation.
Altered vascular tone and cerebral blood flow (CBF)	Ameliorating neurotransmitter dysfunction	Acetylcholinesterase inhibitors (AChEIs), for example donepezil, rivastigmine, velnacrine. Glutamatergic pathway activation, for example memantine.
